# Regulation and tumor-suppressive function of the miR-379/miR-656 (C14MC) cluster in cervical cancer

**DOI:** 10.1002/1878-0261.13611

**Published:** 2024-02-23

**Authors:** Sriharikrishnaa Srinath, Padacherri Vethil Jishnu, Vinay Koshy Varghese, Vaibhav Shukla, Divya Adiga, Sandeep Mallya, Sanjiban Chakrabarty, Krishna Sharan, Deeksha Pandey, Aniruddha Chatterjee, Shama Prasada Kabekkodu

**Affiliations:** 1 Department of Cell and Molecular Biology, Manipal School of Life Sciences, Manipal Academy of Higher Education, India; 2 Department of Bioinformatics, Manipal School of Life Sciences, Manipal Academy of Higher Education, India; 3 Center for DNA Repair and Genome Stability (CDRGS), Manipal Academy of Higher Education, India; 4 Department of Radiotherapy Oncology, Kasturba Medical College, Manipal, India; 5 Department of Obstetrics & Gynecology, Kasturba Medical College, Manipal, India; 6 Department of Pathology, Dunedin School of Medicine, University of Otago, Dunedin, New Zealand

**Keywords:** cervical cancer, methylation, miR-379/miR-656 cluster, tumor suppressor

## Abstract

Cervical cancer (CC) is a key contributor to cancer-related mortality in several countries. The identification of molecular markers and the underlying mechanism may help improve CC management. We studied the regulation and biological function of the chromosome 14 microRNA cluster (C14MC; miR-379/miR-656) in CC. Most C14MC members exhibited considerably lower expression in CC tissues and cell lines in The Cancer Genome Atlas (TCGA) cervical squamous cell carcinoma and endocervical adenocarcinoma patient cohorts. Bisulfite Sanger sequencing revealed hypermethylation of the C14MC promoter in CC tissues and cell lines. 5-aza-2 deoxy cytidine treatment reactivated expression of the C14MC members. We demonstrated that C14MC is a methylation-regulated miRNA cluster via artificial methylation and luciferase reporter assays. C14MC downregulation correlated with poor overall survival and may promote metastasis. C14MC activation via the lentiviral-based CRISPRa approach inhibited growth, proliferation, migration, and invasion; enhanced G2/M arrest; and induced senescence. Post-transcriptional regulatory network analysis of C14MC transcriptomic data revealed enrichment of key cancer-related pathways, such as metabolism, the cell cycle, and phosphatidylinositol 3-kinase (PI3K)–*AKT* signaling. Reduced cell proliferation, growth, migration, invasion, and senescence correlated with the downregulation of active *AKT, MYC*, and cyclin E1 (*CCNE1*) and the overexpression of *p16, p21*, and *p27*. We showed that C14MC miRNA activation increases reactive oxygen species (ROS) levels, intracellular Ca^2+^ levels, and lipid peroxidation rates, and inhibits epithelial–mesenchymal transition (EMT). C14MC targets pyruvate dehydrogenase kinase-3 (*PDK3*) according to the luciferase reporter assay. *PDK3* is overexpressed in CC and is inversely correlated with C14MC. Both miR-494-mimic transfection and C14MC activation inhibited *PDK3* expression. Reduced glucose uptake and lactate production, and upregulation of *PDK3* upon C14MC activation suggest the potential role of these proteins in metabolic reprogramming. Finally, we showed that C14MC activation may inhibit EMT signaling. Thus, C14MC is a tumor-suppressive and methylation-regulated miRNA cluster in CC. Reactivation of C14MC can be useful in the management of CC.

## 1 Introduction

Cervical cancer (CC) is a frequently diagnosed gynecological malignancy in several low-income, middle-income, and developing countries [1]. In 2020, CC contributed to 604 127 cases and 341 831 deaths globally. India (21%) and China (18%) contributed 39% of all cases and 40% of deaths due to CC [2]. Despite the availability of HPV vaccines and screening programs, CC accounts for the highest number of cancer cases among Asian countries [3]. The detection of cancer at an advanced stage and a lack of understanding at the molecular level play key roles in the high incidence and mortality of CC [4]. This highlights the need to understand the molecular players involved in promoting early disease progression and cancer progression toward improved disease management [5].

Small noncoding RNAs (miRNAs) regulate the expression of their target genes to control many physiological and pathological processes [6]. Aberrant miRNA expression is one of the earliest changes promoting carcinogenesis due to its potential to target multiple protein-coding and noncoding genes [7]. We and others have reported abnormal expression of miRNAs in CC [8,9]. Depending on the tissue and expression status of target genes, miRNAs may act as either tumor suppressors or oncogenes [10]. Hence, measuring aberrant miRNA expression can be exploited as a reliable diagnostic and prognostic marker in CC [11]. Interestingly, a large percentage of human miRNAs occur as a discrete group known as miRNA clusters [12]. Most large miRNA clusters contain internal regulators and promoters that drive and fine-tune the expression of members of the miRNA cluster [13,14]. miRNA clusters consist of multiple members that can regulate many genes, leading to simultaneous and stoichiometric regulation of genes involved in functionally related pathways [15,16]. However, the regulation and biological function of large miRNA clusters in CC have not been fully elucidated.

The second largest human miRNA cluster, microRNA-379/656 (C14MC), is driven by a single promoter and multiple internal regulators. This gene is located at 14q32.31, spans ~ 45 kb in size, and coregulates and coexpresses 42 miRNAs [17]. The 14q32.31 locus contains imprinted genes (*DLK1, RTL1, MEG3, MEG8*, and *DIO3*), C/D-type small nucleolar RNAs, and more than 50 miRNA-encoding genes [18]. C14MC expression is limited mainly to the placenta and tissues of epithelial origin and is expressed from maternally inherited copies [19]. Several human diseases and malignancies exhibit aberrant C14MC expression. C14MC members exhibit tumor-suppressive and oncogenic characteristics depending on the cell and tissue type [17]. In a few cancers [16,20,21], abnormal expression of C14MC members is correlated with poor prognosis and aggressiveness [22]. Genetic and epigenetic aberrations contribute to the abnormal expression of C14MC in various cancers. In addition to the promoter, internal regulators play a critical role in regulating C14MC expression [23,24].

We and others have reported that members of the C14MC are abnormally expressed and may have a tumor-suppressive effect on CC [25,26]. For instance, miR-379 and miR-411 downregulation was correlated with tumor size, FIGO stage, and metastasis [27]. C14MC targets several tumor-promoting genes (*DLUE1, HOXA13, MAPK1, BMI1, CRKL, STAT3, STK17A, MTF2, MACC1, ZEB2, SGK3*, and *FGF7*) to inhibit growth, proliferation, migration, invasion, and apoptosis. The possible interaction between C14MC members and HPV-encoded proteins has been reported in CC [28]. However, the role of the entire C14MC in the CC has yet to be understood, as previous studies have focused on understanding the role of individual members of the C14MC rather than the whole cluster. Hence, the present study aimed to investigate the regulation and biological function of C14MC in CC. We detected C14MC downregulation by analyzing its expression in clinical samples and CC cell lines. We demonstrated that C14MC is a methylation-driven miRNA cluster in CC. C14MC activation had tumor-suppressive effects on CC. Clinically, profiling C14MC expression may be helpful as a diagnostic and prognostic indicator in CC.

## 2 Materials and methods

### 2.1 Cell culture

We procured the cell lines from ATCC, USA and cultured human CC cell lines (HeLa, RRID:CVCL_0030; SiHa, RRID:CVCL_0032; and CaSki, RRID: CVCL_1100) using standard protocol available at www.atcc.org. The cell lines were maintained in media (SiHa and HeLa: DMEM; CaSki: RPMI) supplemented with 10% fetal bovine serum. The cells were cultured without serum for 48 h for senescence induction [29]. All the experiments were performed with mycoplasma-free cells. Authenticity of the cell lines was confirmed using GenePrint-10 System (Promega, Madison, WI, USA). In brief, 10 ng of DNA isolated from cell lines were subjected to PCR amplification using GenePrint^®^ 10 5X Primer Pair Mix and GenePrint^®^ 10 5X Master Mix according to manufacturer’s instruction. The amplified fragments were analyzed using 3130xl Genetic Analyzer. Cell lines with ≥ 90% match is considered for experiment (Table S1).

### 2.2 Clinical samples

We obtained normal cervical epithelium (NCE) and squamous cell carcinoma (SCC) samples from the Department of Obstetrics and Gynecology and the Department of Radiotherapy and Oncology, Kasturba Medical College, Manipal, India upon approval from the Institutional Ethical Committee at Kasturba Hospital, Manipal (IEC 763/2016). Informed written consent forms were taken from the patients/individuals who visited the hospital before collecting the samples. The samples were collected within the time duration of December 2016 to December 2021.The study adhered to the principles and guidelines of the Declaration of Helsinki. Normal samples were collected from cancer-free participants who visited Kasturba Medical College, Manipal, for regular cervical screening. The study included freshly diagnosed, histopathologically confirmed SCC patients with no prior history of cancer treatment. DNA and RNA were extracted from tissues and cell lines using a DNA extraction kit and TRIzol reagent (Thermo Fischer Scientific, Waltham, MA, USA) [9,25,30]. All experiments were conducted in duplicate, and the resulting data are presented as mean ± standard deviation (SD).

### 2.3 Methylation and demethylation studies

We used the MethPrimer tool to design primers [31]. We designed primers for the region predicted to be a C14MC promoter by Fantom 5 (https://fantom.gsc.riken.jp/5/) for methylation analysis via bisulfite Sanger sequencing. For methylation analysis, the DNA modified using the EZ DNA Methylation Kit (Zymo Research, Irvine, CA, USA) was amplified using primers (FP: GGGGTATTTTTGGTTAAGG TGG and RP:CTCRCCAAACAAAATAAATTC) in a thermocycler (Eppendorf, Hamburg, Germany) under the following cycling conditions: 95 °C for 5 min; 34 cycles of 95 °C for 45 s, 57.5 °C for 30 s and 72 °C for 1 min; and one cycle of 72 °C for 5 min. We used the BigDye Terminator v3.1 (Thermo Fischer Scientific) kit and 3130xl Genetic Analyzer to sequence the agarose gel-purified PCR products. The trace files were manually analyzed using the Chromas (https://technelysium.com.au/wp/) tool to calculate the extent of methylation at each CpG site [9]. We exposed subconfluent SiHa, HeLa, and CaSki cells to 5 μm 5-aza-2′-deoxycytidine for 72 h. Subsequently, DNA and RNA were extracted and subjected to methylation profiling and expression analysis using bisulfite Sanger sequencing and qRT–PCR, respectively [25,32].

### 2.4 Promoter characterization

We cloned the predicted promoter region (chr14:101 291 001–101 292 434) of C14MC into the pGL3-Basic vector to generate the pGL3-C14MC construct. We artificially methylated the pGL3-C14MC construct using M.SssI (NEB, Ipswich, MA, USA) [33]. We generated 3 deletion constructs of full-length pGL3-C14MC by restriction digestion and ligation. We verified the construct using DNA sequencing and restriction digestion. Individual constructs were cotransfected with SV40 using Lipofectamine (Thermo Fischer Scientific). We measured promoter activity with a dual luciferase assay kit (Promega) 48 h post-transfection [33].

### 2.5 qRT–PCR analysis

We used a High-Capacity cDNA Reverse Transcription Kit (Applied Biosystems, Waltham, MA, USA) for cDNA synthesis. qRT–PCR was performed for the hsa-miR-376c-3p, hsa-miR-494-3p, hsa-miR-495-3p, and pyruvate dehydrogenase kinase 3 (*PDK3*) genes using the TaqMan assay. RNU6B and β-actin were used as internal controls. The experiment used a 7500 Fast real-time PCR (Thermo Fischer Scientific) instrument. The data were analyzed using the 2^−ΔΔCT^ formula [25].

### 2.6 Small RNA sequencing

We used previously published small RNA sequencing data to analyze miR-379/656 expression [25]. We used the CAP-miRSeq tool for processing, analyzing, and identifying the differentially expressed miRNAs. We manually screened the differentially expressed miRNAs generated by small RNA sequencing to determine C14MC expression in CC [34].

### 2.7 Public dataset analysis

We searched for C14MC expression in The Cancer Genome Atlas Cervical Squamous Cell Carcinoma and Endocervical Adenocarcinoma (TCGA-CESC) [35] dataset using the TACCO tool [36]. We used *P* < 0.05 and |logFC| ≥ 1 as criteria for identifying the differentially expressed miRNAs. The diagnostic significance of C14MC expression was computed using the random forest algorithm. The prognostic significance of the C14MC cluster was analyzed using the miRNOME tool [37]. The C14MC cluster targets were predicted using TargetScan [38]. The C14MC members were manually searched in PubMed and Google Scholar to identify their association with metastasis. We used the STRING online tool to construct a protein–protein interaction network (PPIN) of C14MC target genes [39]. With respect to the PPIN, we predicted the top ten hub genes using the cytoHubba tool [40]. Gene Ontology (GO) and pathway enrichment analyses were carried out using ShinyGO [41].

### 2.8 Establishment of C14MC-activated CaSki cells

The promoter sequence of C14MC was subsequently sent to Applied Biological Materials, Canada, to design and clone the guide RNAs in a dCas9-SAM lentiviral vector. The scrambled sgRNA CRISPR lentivector served as a control. Three sgRNAs were combined with a third-generation packaging mixture (Applied Biological Materials, Richmond, Canada) and transfected into HEK293T cells using Lipofectamine™ 3000 (Thermo Fischer Scientific). Viral soups were collected, passed through a 0.45-μm sieve, and used to transduce CaSki cells. The transduced cells were exposed to G418 (400 μg·mL^−1^) for 2 weeks to select C14MC-activated clones. Successful endogenous activation was confirmed by targeted DNA sequencing and upregulation of C14MC cluster member expression compared to that in scrambled-transfected cells, as determined by qRT–PCR [42]. In a separate set of experiments, SiHa cells were transiently transfected with a negative control, hsa-miR-494-3p or hsa-miR-409-3p mimics for subsequent experiments to further confirm the findings of C14MC activation [26].

### 2.9 Transcriptomic analysis

RNA-seq was carried out according to the workflow recommended by Life Technologies (Carlsbad, CA, USA) to identify the C14MC-responsive genes [43]. rRNA was depleted using the RiboMinus Eukaryote Kit v2, and mRNA libraries were generated using the Ion Total RNA-Seq V2 Kit. Emulsion PCR and enrichment were carried out using the Ion PI Template OT2 200 Kit v3 and Ion OneTouch ES. We used an Ion PI Chip Kit v2 and an Ion Proton System for sequencing. The DEGs were identified using the DESeq2 tool [44].

### 2.10 Actin-phalloidin staining

Scrambled and C14MC-activated cells were cultured on a glass-bottom Petric plate to 70–80% confluence (IBIDI, Bayern, Germany). Cells fixed with 4% paraformaldehyde were incubated with TRITC-labeled actin-phalloidin and stained with Hoechst. The cells were imaged with a DMi8-SP8 laser scanning confocal microscope (Leica Microsystems, Wetzlar, Germany, 100× magnification) and analyzed with LAXs software to measure filopodia length [45].

### 2.11 Colony formation assay

We cultured scrambled and C14MC-activated cells (500 cells, 21 days) in complete medium in a six-well plate. After media removal and brief washing with PBS, the colonies were stained with 0.5% crystal violet stain in methanol (15 min). Excess stain was added, after which the sections were rinsed with distilled water and imaged. Colonies containing more than 50 cells as visualized through bright field microscopy were included [29].

### 2.12 Proliferation and growth curve analysis

Scrambled and C14MC-activated cells (1 × 10^4^ cells) were plated in 35-mm Petri dishes in duplicate for 5-day growth curve analysis [33]. After trypan blue staining, the trypsinized cells were counted in a hemocytometer at the end of day 1, day 3, and day 5 for cell doubling time calculation.

### 2.13 Migration assay

C14MC-activated and scrambled cells were subjected to serum starvation for 24 h. A sterile microtip was used to make a wound in the center of the plate. Fresh medium containing 10% FBS was added, and cells that had migrated into the wound region were monitored using a CK-41 microscope (Olympus, Tokyo, Japan) connected to a camera [29,45].

### 2.14 2D agarose spot invasion assay

A six-well plate with an agarose spot containing EGF (50 ng·mL^−1^) in serum-free DMEM was loaded with C14MC-activated and scrambled cells (3 × 10^5^). The concentrations of agarose and EGF were 0.5% and 50 ng·mL^−1^ respectively. The cells that entered the agarose spot were considered invading cells [46]. The cells invading the agarose spot were imaged at 10× magnification using a Rolera Emc2 camera and CKX-41 microscope (Olympus). The cells that penetrated the agarose spot were manually scored from five random fields to determine the degree of invasion.

### 2.15 Cell cycle assessment

C14MC-activated and scrambled control cells were expanded to 50–60% confluence and maintained for 24 h without serum. After that, the cells were grown for 24 h in complete medium, trypsinized, and stained with a mixture of RNase (10 mg·mL^−1^; Sigma, St. Louis, MO, USA) and propidium iodide (50 mg·mL^−1^; Sigma) [33]. The data were acquired and examined using a FACSCalibur and CellQuest software (BD Biosciences, Franklin Lakes, NJ, USA).

### 2.16 Senescence assay

We used senescence-associated beta gal (SA-Gal) staining to quantify the extent of senescence in 48 h serum-starved C14MC-activated and scrambled control cells [39]. We used a DP80 camera and a BX51 microscope to capture the images. We counted the beta-gal-positive cells in five random fields (Olympus).

### 2.17 C14MC target validation

A 1389 bp 3′-UTR of *PDK3* was amplified using primers (FP: TATGCTAGCAATTAGCTCCTCTCCC ACC) and (RP: TGCTCTAGATAAATAGACAC AGTCCCCAC) containing the target sequence of miR-494, which was subsequently cloned and inserted into the pmirGLO vector (Promega). We performed a Dual-Glo^®^ Luciferase Assay (Promega) using the lysate prepared from SiHa cells cotransfected with the 3′-UTR construct and the miR-494 mimic using Lipofectamine 3000 (Thermo Fischer Scientific). Luminescence was measured with a single-channel luminometer (Berthold, Bad Wildbad, Germany) [25].

### 2.18 Western blotting

The proteins were extracted from C14MC-activated or scrambled cells in RIPA buffer supplemented with a protease inhibitor cocktail. Approximately 30–60 μg of protein was separated by 10% SDS/PAGE and transferred to a nitrocellulose membrane. The membranes were then blocked with 5% non-fat dry milk prepared in TBST, washed, and incubated with various concentrations of 1° antibodies, such as anti-*PDK3*, anti-*p16*, anti-*p21*, anti-*p27*, p-*AKT*, total-*AKT*, anti-*c-MYC*, anti-*CCNE*1, anti-*CDH1*, anti-CDH2, anti-*VIM*, anti-SNAIL1, and anti-ACTB (Table S2). The TBST-washed membranes were probed with HRP-conjugated 2° antibodies. We used SuperSignal™ West Pico Chemiluminescent Substrate (Thermo Fischer Scientific) to develop and visualize the proteins. Western blotting images were obtained using an Image Quant LAS 4000 gel documentation system [47].

### 2.19 ROS and Ca^2+^ estimation

C14MC-activated and scrambled cells were incubated with DCFDA (1 μg·mL^−1^) and Fluo-3AM (1 μg·mL^−1^) for 30 min at RT to measure ROS and Ca^2+^, respectively. For Ca^2+^ measurements, the cells were incubated with Fluo-3AM and 20% pluronic F127. After removing the stain, the cells were washed with PBS and HBSS, and images were captured using a DMi8-Sp8 confocal microscope (DCFDA: excitation at 498 nm and emission at 522 nm; Fluo-3AM: excitation at 506 nm and emission at 528 nm) [48].

### 2.20 Glucose uptake

C14MC-activated and scrambled cells (200 000 cells) were cultured in serum-starved medium for 48 h in a 6-well plate. At 12-h intervals, conditioned medium was collected for up to 48 h. The glucose in the conditioned medium was quantified calorimetrically (absorbance: 505 nm) using a Varioskan multimode reader (Thermo Fischer Scientific) and a glucose estimation kit from Agape, India. The amount of glucose consumed was calculated by subtracting the OD at each time interval from that at 0 h. The data were normalized against the total protein concentration [48].

### 2.21 Lactate production

C14MC-activated and scrambled cells were serum-starved and lysed using lysis buffer (20 mM Tris/HCl pH 7.5, 150 mM NaCl, 1 mM EDTA, and 1% Triton X-100). The supernatant was collected by centrifugation at 8000 ***g*** for 10 min. The supernatant was incubated with 50 mM PIPES buffer (pH 7.5) at RT for 10 min. Using a VarioSkan reader, we measured the absorbance at 505 nm and normalized the value to the total protein concentration [48].

### 2.22 Lipid peroxidation assay

The thiobarbituric acid (TBA) method was utilized to measure the rate of lipid peroxidation in C14MC-activated and scrambled cells. Serum-starved cells were washed with prechilled PBS and lysed using lysis buffer (20 mM Tris/HCl (pH 7.5), 150 mM NaCl, 1 mM EDTA, and 1% Triton X-100). The supernatant was collected by centrifuging at 8000 ***g*** for 10 min. PBS (pH 7.4) was added to the supernatant, which was subsequently incubated for 1 h at 37 °C. We added 20% TCA and 1% thiobarbituric acid, incubated the mixture in a boiling water bath for 30 min, and centrifuged the mixture at 10 000 rpm for 15 min. The color developed was subsequently measured at 532 nm using a Varioskan instrument [49]. We used the total protein content for normalization of the data.

### 2.23 Statistical analysis

A *P* value < 0.05 according to Student’s *t* test and ANOVA was considered to indicate statistical significance. The data are presented as the means ± SEMs. The experiments were performed in duplicate and repeated three times.

## 3 Results

### 3.1 CC shows downregulation of C14MC

**Fig. 1:**
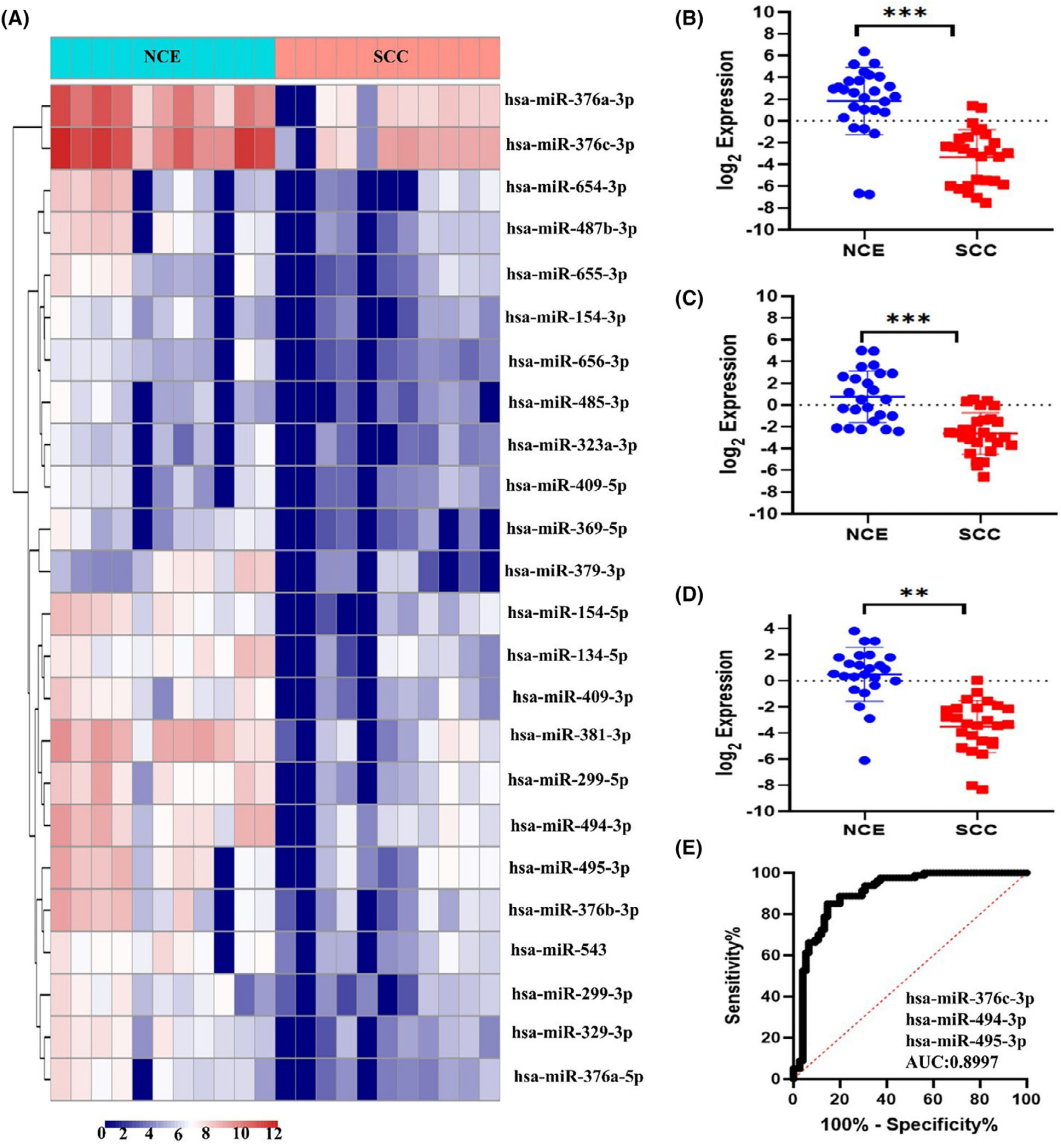
Analysis of C14MC expression in normal and CC samples. (A) Heatmap of unsupervised hierarchical clustering of the expression of C14MC members. (B–D) qRT–PCR analysis of C14MC miRNA family members in NCE (*n* = 25) and SCC (*n* = 25) samples showing downregulation of hsa-miR-376c-3p, hsa-miR-494-3p, and hsa-miR-495-3p in SCC compared with NCE. (E) The receiver operating characteristic curve (ROC) curve showed an AUC of 0.899 (95% CI = 0.8997). Sum of the expression of hsa-miR-376c-3p, hsa-miR-494-3p, hsa-miR-495-3p taken for plotting ROC. The error bar represent mean ± SD of duplicate experiments performed in triplicates. Statistical significance was determined by unpaired Student’s *t*-test (***P < 0.0005; **P < 0.01).

The analysis of small RNA sequencing data showed a global downregulation of C14MC (24 out of 42 members) in SCC compared with normal samples (Fig. 1A). However, small RNA sequencing did not detect the expression of the remaining miRNAs. Analysis of the TCGA-CESC dataset via the LinkedOmics database [50] revealed that 36 miRNA members of C14MC were downregulated in CC (Fig. S1). The results of small RNA sequencing and analysis of the TCGA-CESC dataset confirmed the expression of C14MC members, such as hsa-miR-376c-3p, hsa-miR-494-3p, and hsa-miR-495-3p, by qRT–PCR. All three miRNAs selected for validation were downregulated more than twofold according to our NGS data and were expressed at different locations within the C14MC locus. The qRT–PCR analysis showed downregulation of hsa-miR-376c-3p (Fig. 1B), hsa-miR-494-3p (Fig. 1C), and hsa-miR-495-3p (Fig. 1D) in SCC. Sensitivity and specificity analyses revealed an area under the curve (AUC) (95% CI = 0.8997) of 0.89 (Fig. 1E). The TCGA-CESC and miRNOME analysis data are shown in (Tables S3 and S4), respectively.

### 3.2 Characterizing the C14MC promoter

**Fig. 2:**
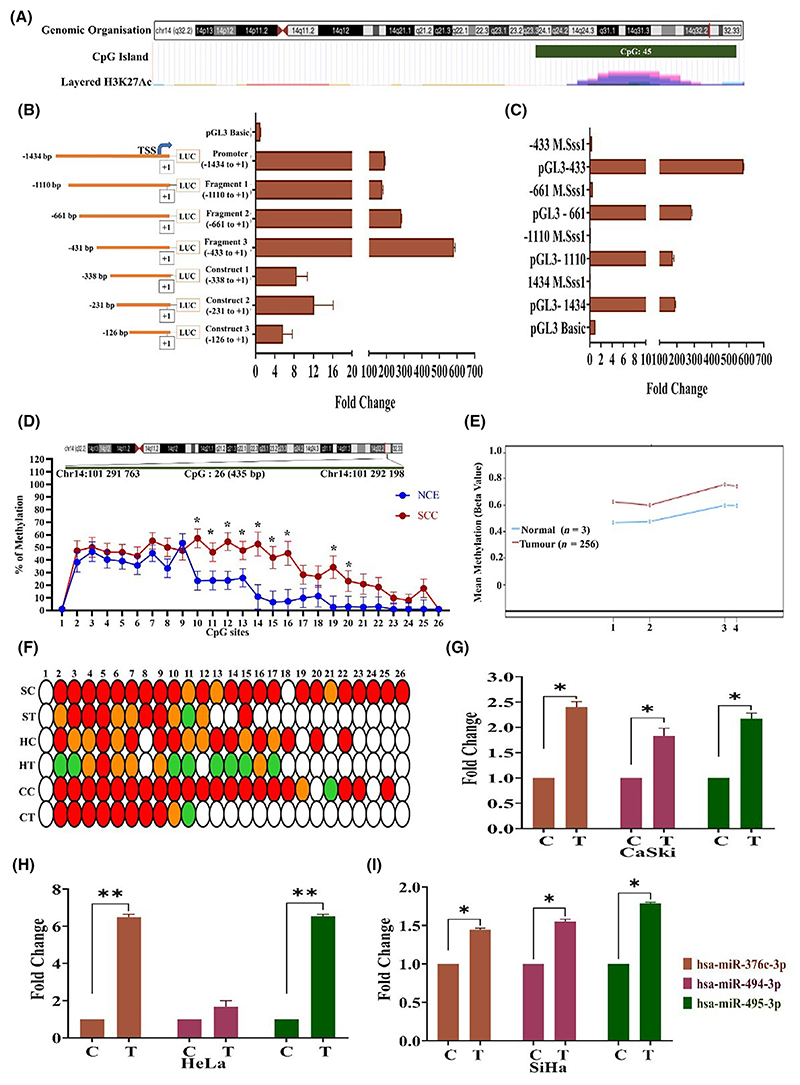
C14MC is a methylation-driven miRNA cluster in CC. (A) Genomic organization of the upstream region of C14MC. (B) The luciferase reporter assay suggested that −1434 to +1 had robust promoter activity and may contain the C14MC promoter. (C) *In vitro* methylation of −1434 to +1 significantly decreased promoter activity, suggesting that the C14MC promoter may be driven by methylation. (D) Bisulfite Sanger sequencing (BGS) of 26 CpG sites within the predicted C14MC promoter. Values in the graph represents mean ± SEM by two-way ANOVA (*P < 0.05). (E) TCGA-CESC data showing hypermethylation of CpG (1-cg20952167, 2-cg12967319, 3-cg04304932, 4-cg04576764) sites upstream of C14MC. (F) BGS of CC cell lines before and after 5-Aza 2 Deoxy cytidine treatment. (White: 0–25% methylation, Green: 26–50% methylation, Orange: 51–75% methylation, Red: 76–100% methylation) (SC, SiHa Control; ST, SiHa Treated; HC, HeLa Control; HT, HeLa Treated; CC, CaSki Control; CT, CaSki Treated). (G–I) Re-expression of hsa-miR-376c-3p, hsa-miR-494-3p, and hsa-miR-495-3p upon 5-Aza 2 Deoxy cytidine treatment in CaSki, HeLa, and SiHa cells. Values in the graph represents mean ± SD. Statistical significance was determined by unpaired Student’s *t*-test. *Denotes *P* < 0.05, ** denotes *P* < 0.01. C: untreated, T: treated with 5-Aza 2 Deoxy cytidine. Experiments are performed in duplicate and repeated three times.

We predicted the promoter of C14MC by sequence and regulatory feature analysis using the FANTOM5 tool (https://fantom.gsc.riken.jp/5/). The genomic coordinate (chr14:101 291 001–101 292 434) located within the MEG3 region contained a CpG island, was enriched in the active histone marker H3K27ac (Fig. 2A) and contained multiple cis-regulatory elements along with the p1@MEG3 precursor miRNA promoter. The region also contains open chromatin regions, as shown by FAIRE and DNaseI hypersensitivity analysis and the SwichGear genomics transcription start site. These analyses suggested that the region is likely to be a putative promoter region of C14MC. We subsequently cloned the 1434 bp (chr14:101 291 001–101 292 434; Fig. 2B) region in the pGL3-Basic vector and performed a dual luciferase reporter assay to confirm the promoter activity of the region. In addition, we generated 3 deletions of the full-length construct by restriction digestion, namely, fragment 1 (+1 to −437 bp), fragment 2 (+1 to −665 bp) and fragment 3 (+1 to −1114). The full-length promoter construct showed robust promoter activity (Fig. 2B). Among the three deletion constructs, fragment 3 showed significantly greater promoter activity (Fig. 2B). DNA methylation is perhaps the strongest genomic regulator of promoter activity [51,52]. Therefore, we next performed a dual-luciferase reporter assay using artificially methylated full-length and deletion constructs of the C14MC upstream region to evaluate the impact of methylation on promoter activity. DNA methylation significantly decreased promoter activity compared to that of the unmethylated constructs (Fig. 2C). *MEF*2 was shown to be a C14MC regulator in rat neurons, oligodendrogliomas and muscle cells [22]. Hence, we analyzed the expression of four isoforms of *MEF*2 (*MEF*2A, *MEF*2B, *MEF*2C and *MEF*2D) in the TCGA-CESC cohort using the OncoDB tool [53]. All four isoforms of *MEF*2 were significantly downregulated and hypermethylated in TCGA-CESC cancer samples (*n* = 304) compared with normal (*n* = 22) samples (Fig. S2A–D). Interestingly, the expression of C14MC members and *MEF*2 isoforms was co-downregulated in the TCGA-CESC cohort. The prediction of the transcription factor-binding site at chr14:101 291 001–101 292 434 region using the PROMO tool (https://alggen.lsi.upc.es/cgi-bin/promo_v3/promo/promoinit.cgi?dirDB=TF_8.3) revealed the binding of *MEF*2A (Fig. S2E). The upstream region of C14MC analyzed in the present study is highly likely to represent the C14MC promoter and regulatory features, whose activity is controlled by DNA methylation levels.

### 3.3 SCC contains a hypermethylated C14MC promoter

C14MC members are downregulated in a few malignancies due to hypermethylation [22]. Bisulfite Sanger sequencing suggested that among the 26 CpG sites analyzed, CpG sites 10–18 exhibited significant hypermethylation in SCC samples compared with normal samples (*n* = 25 each; Fig. 2D). Analysis of the TCGA-CESC patient cohort revealed significant hypermethylation [(1-cg20952167, 2-cg12967319, 3-cg04304932, 4-cg04576764)] in tumors with average beta values of 0.68 and 0.53 in tumor and normal samples, respectively (Fig. 2E). The C14MC promoter was highly methylated in SiHa, HeLa, and CaSki cells, and treatment with 5-azacytidine significantly reduced the methylation levels at multiple CpG sites. In addition, treatment with 5-azacytidine restored the expression of members of the C14MC cluster, namely, hsa-miR-376c-3p, hsa-miR-494-3p, and hsa-miR-495-3p (Fig. 2F–H), as evaluated by qRT–PCR.

### 3.4 Clinical significance of C14MC downregulation

We tested the diagnostic and prognostic significance of C14MC downregulation using the TCGA-CESC dataset. We constructed a pathological classification and prognosis prediction model using significantly differentially expressed C14MC miRNAs in the TCGA-CESC cohort by implementing the random forest algorithm in the TACCO tool (http://tacco.life.nctu.edu.tw/). The sensitivity and specificity of C14MC expression for predicting the T stage were 1 and 0.87, respectively (Fig. S3A). The sensitivity and specificity of C14MC for predicting the spread of cancer to nearby tissues (N stage) were 0.98 and 0.77, respectively (Fig. S3B). The sensitivity and specificity of the C14MC for predicting metastasis (M0 vs. M1) were 1 and 0.8, respectively (Fig. S3C). The prognostic prediction model showed that C14MC expression significantly affected the overall survival (OS) and disease-free survival (DFS) of CC patients (Fig. S3D,E). Thus, C14MC expression can be useful for categorizing patients into high-risk and low-risk groups. The sensitivity and specificity of the prognostic model for OS and DFS were 0.88 and 0.97 and 0.91 and 0.92, respectively. A literature search of C14MC members using PubMed and Google Scholar identified hsa-miR-381 [54], hsa-miR-329 [55], hsa-miR-376c [56], hsa-miR-379 [57], and hsa-miR-494 [58] as metastasis inhibitors in CC.

### 3.5 Identification of the C14MC target network in CC

We assessed the potential post-transcriptional regulatory networks of C14MC using an *in silico* approach. We selected all the C14MC members and predicted the targets of each miRNA in the TCGA-CESC dataset. Since C14MC members exhibit global downregulation in CC, we included the C14MC target genes that were significantly upregulated in the TCGA-CESC dataset for protein–protein interaction network (PPIN) construction and to identify enriched gene ontology terms and pathways. We identified 542 predicted upregulated targets of C14MC in the TCGA-CESC cohort. GO analysis of 542 overexpressed genes using ShinyGO revealed that cell division and the cell cycle were enriched biological processes. The top ten pathways associated with genes exhibiting significant enrichment in the KEGG pathway database were involved in cancer-related microRNAs, p53 signaling, prostate cancer, the cell cycle, small cell lung cancer, Epstein–Barr virus infection, cancer pathways, PI3K–AKT signaling, and human papillomavirus infection (Fig. S4A–D). Most of the pathways identified in our study were well-established drivers of CC. The PPIN of 542 overexpressed genes generated a network of 449 nodes and 2777 edges with a PPI enrichment p value < 1.0e-16 (Fig. S5A). The screening of PPINs using the cytoHubba tool revealed *CDK1, AURKA, BUB1, BIRC5, TTK, CCNB1, CEP55, MAD2L1, TOP2A*, and *KIF2C* as the top 10 hub genes of the network (Fig. S5B) and its Gene Ontology (Fig. S6A–D).

### 3.6 Ectopic expression of C14MC inhibited the growth, proliferation, migration, and invasion of CaSki cells

**Fig. 3:**
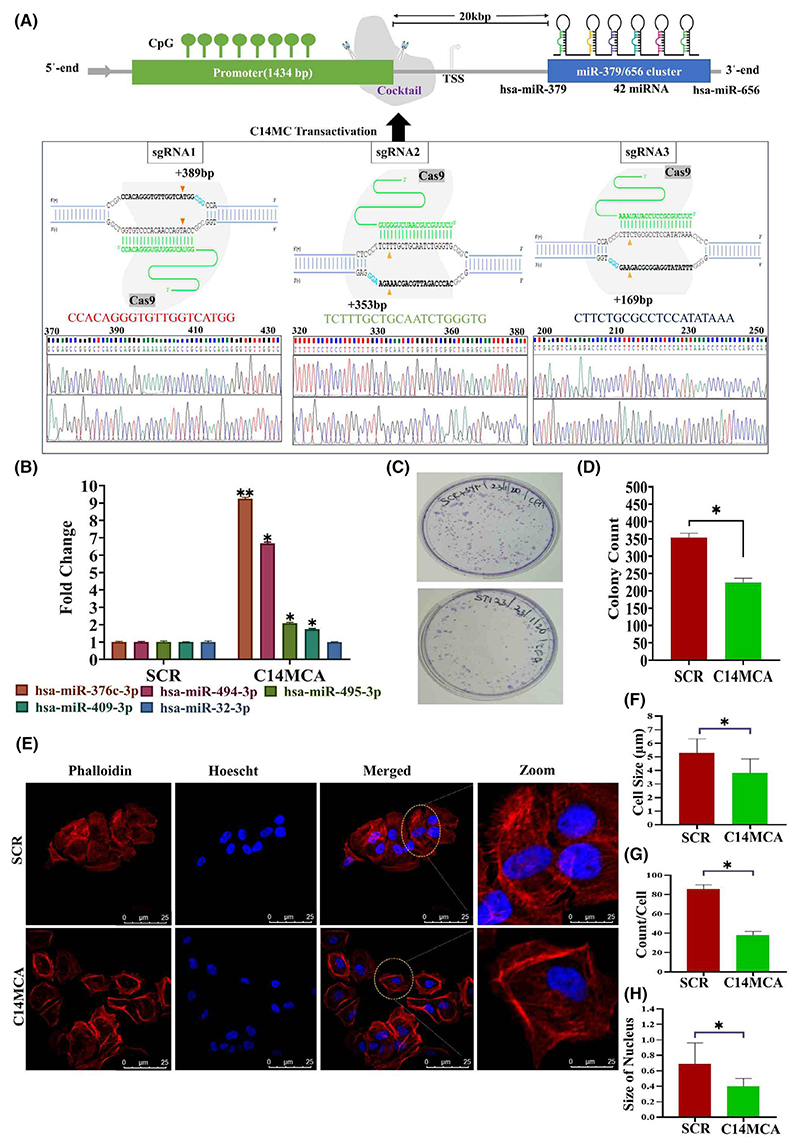
Activation of C14MC inhibits tumor growth and induces morphological changes in CaSki cells. (A) Schematic diagram showing the region used to activate C14MC and its confirmation by Sanger sequencing. (B) qRT–PCR showing the upregulation of hsa-miR-376c-3p, hsa-miR-494-3p, hsa-miR-495-3p, and hsa-miR-409-3p in response to C14MC activation in CaSki cells. hsa-miR-32-3p is not a member of the C14MC and was used as a control in this study. C14MC activation did not upregulate hsa-miR-32-3p expression. Values in the graph represent mean ± SD of duplicates. Statistical significance was determined by unpaired Student’s *t*-test; ***P < 0.005, **P < 0.01. (C, D) C14MC activation inhibited the growth of CaSki cells, as determined by a colony formation assay. (E) Morphological analysis of C14MC-activated cells by Actin-phalloidin staining and confocal microscopy. (F–H) C14MC activation reduced the cell size, number of filopodia, and number of nuclei. The images were captured at 63× magnification and Image Scale – 25 μm. Values in the graph represent mean ± SD of duplicates. Statistical significance was performed by unpaired Student’s *t*-test, images were quantified using IMAGEJ software, *P < 0.05. Experiments are performed in duplicate and repeated three times.

**Fig. 4:**
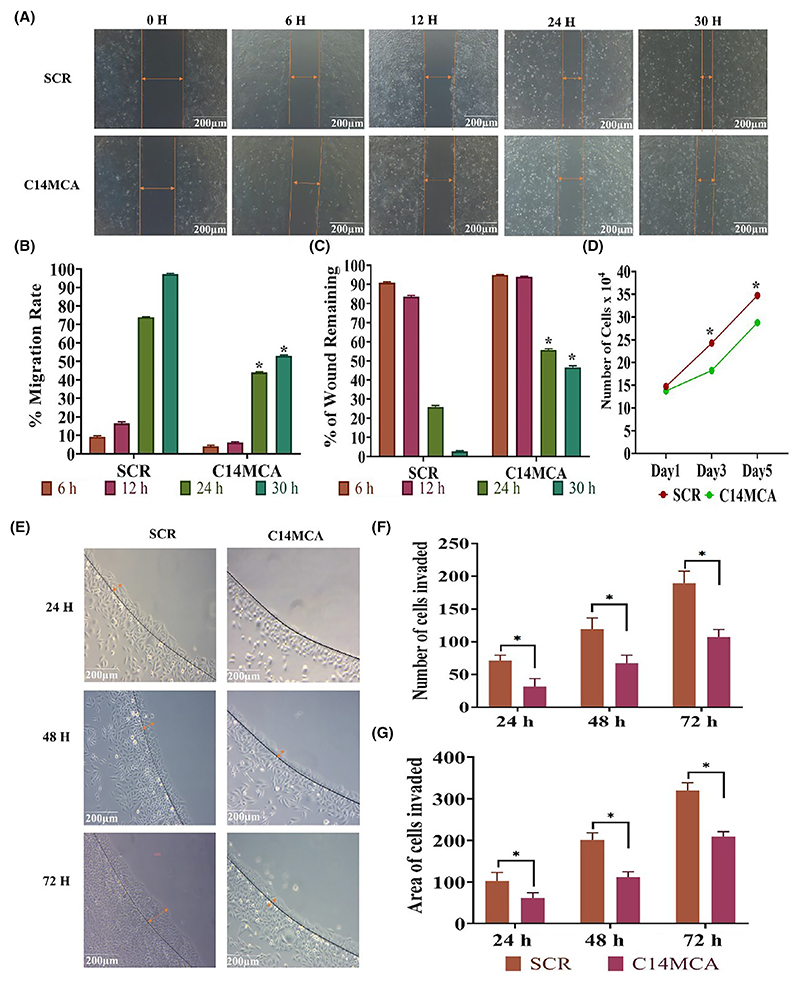
Activation of C14MC inhibits the proliferation, migration, and invasion of CaSki cells. (A) Representative images of the scratch assay. Scale bar: 200 μm. Orange arrow indicates the area of cells migrated. (B, C) Quantitative analysis of the migration rate and percentage of wounds remaining on C14MC-activated and scrambled CaSki cells. (D) Compared with scrambled CaSki cells, C14MC-activated CaSki cells exhibited a significantly lower proliferation rate and longer cell doubling time. (E) Representative images of the agarose spot invasion assay (Image Scale-200 μm). Orange arrow indicates the area of cells invaded. (F) Quantitative analysis of cells invading agarose spots. (G) Quantitative analysis of the depth of invasion. Values in the graph represent mean ± SD of duplicates. Statistical significance was tested by unpaired Student’s *t*-test, images were quantified using IMAGEJ software, *P < 0.05. Experiments are performed in duplicate and repeated three times.

We activated the C14MC cluster by using the CRISPR/Cas9 genome editing approach. We performed qRT–PCR for randomly selected members of C14MC to confirm its activation (Fig. 3A). We observed that hsa-miR-376c-3p (7-fold), hsa-miR-494-3p (6-fold), hsa-miR-495-3p (2-fold), and hsa-miR-409-3p (2-fold) were significantly elevated in C14MC-activated cells compared with scrambled vector-transfected control cells (Fig. 3B). We did not observe any change in the expression of hsa-miR-32-3p, a non-member of the C14MC cluster, as determined by qRT–PCR (Fig. 3B). The results of the colony formation assay showed that C14MC activation significantly inhibited growth, as demonstrated by the reduction in colony number compared with that in cells treated with scrambled shRNA (Fig. 3C,D). C14MC activation significantly reduced CaSki cell proliferation according to a five-day growth curve analysis (Fig. 4D). The scratch and agarose spot invasion assay results showed that C14MCs reduced the migration (Fig. 4A–C) and invasion (Fig. 4E–G).

### 3.7 C14MC activation induces morphological changes, cell cycle arrest, and senescence

**Fig. 5:**
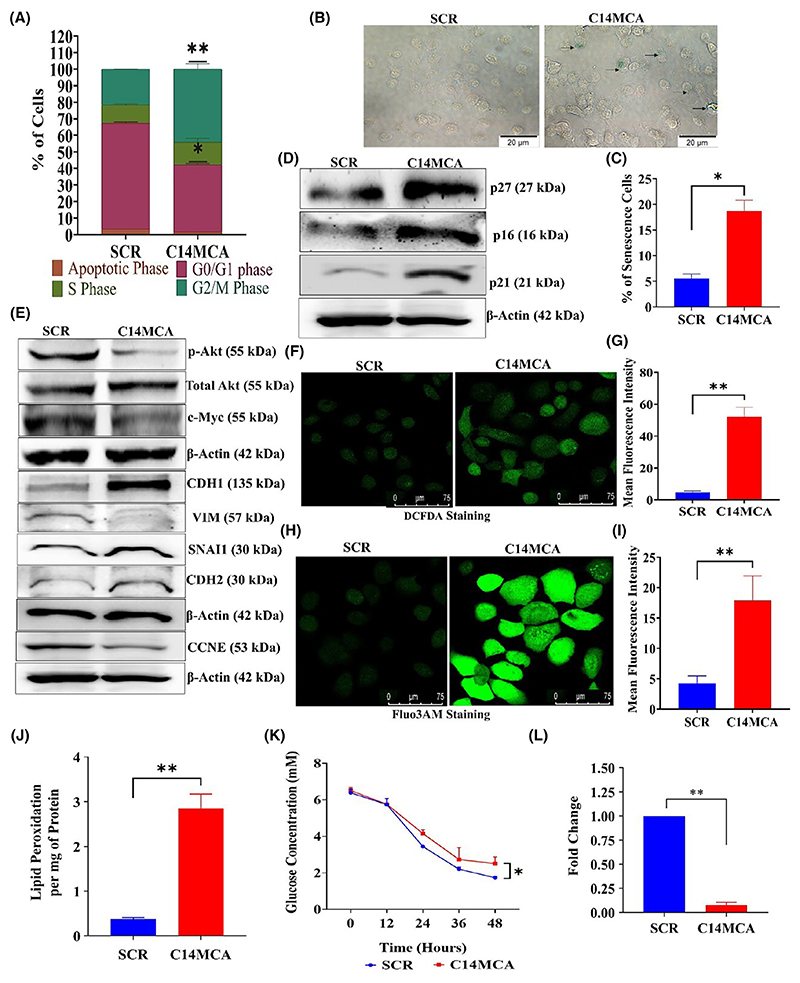
C14MC activation induces G2/M arrest and senescence and negatively regulates EMT in CaSki cells. (A) Bar graph showing G2/M arrest in CaSki cells in response to C14MC activation. Values in the graph represent mean ± SD of duplicates and statistical significance was performed by unpaired Student’s *t*-test; **P < 0.01, *P < 0.05. (B) C14MC activation significantly increased the number of beta-gal-positive cells, as determined by senescence-associated beta-gal staining (Image Scale – 20 μm). (C) Bar graph showing the quantitative analysis of beta-gal-positive C14MC-activated and scrambled vector-transfected CaSki cells. (D) Western blot showing the upregulation of *p16, p21*, and *p27* in response to C14MC activation in CaSki cells. (E) Western blot images showing upregulation of *CDH1* and downregulation of the active forms of *AKT*1, *c-MYC, VIM*, and *CCNE*. (F, G) Representative images of DCFDA-stained CaSki cells activated with C14MC or transfected with a scrambled vector (Image Scale – 75 μm). (H, I) Representative images of CaSki cells stained with Fluo3-AM and C14MC-activated or scrambled vector (Image Scale – 75 μm). (J) C14MC activation increases the lipid peroxidation rate in CaSki cells. (K) Bar graph showing a decrease in glucose uptake in response to C14MC activation. (L) C14MC activation significantly decreased lactate levels compared with those in scrambled vector-transfected CaSki cells. Values in the graph represent mean ± SD of duplicates. Statistical significance was performed by unpaired Student’s *t*-test; **P < 0.01, *P < 0.05. Fluorescence intensities were measured using IMAGEJ software. Experiments are performed in duplicate and repeated three times.

C14MC activation induces significant morphological changes through actin cytoskeletal rearrangements and decreased filopodia (Fig. 3E–H). PI staining and flow cytometry revealed significant G2/M arrest but no significant difference in the percentage of apoptotic cells between the C14MC-activated and scrambled cells (Fig. 5A). We next investigated senescence induction, as there was strong G2/M arrest in response to C14MC activation in CaSki cells. There were significantly more senescence-positive C14MC-activated cells than scrambled CaSki cells (18.7% vs. 4.6%, Fig. 5B, C), as analyzed by senescence-associated beta-gal staining. We observed that C14MC activation enhanced *p16, p21*, and *p27* expression in CaSki cells more than in scrambled cells (Fig. 5D).

### 3.8 Overexpressing C14MC inhibits the active form of *AKT* and *c-MYC*

*AKT* and *c-MYC* are the key frequently activated oncoproteins in CC [59]. Activation of *AKT* and *c-MYC* promotes growth, proliferation, migration, and invasion by regulating cellular metabolism [60]. The results of the western blot analysis showed that the C14MC activation reduced the active form of *AKT* and *c-MYC* without changing the total *AKT* level (Fig. 5E). Thus, the reduced growth, proliferation, migration, invasion and induction of senescence in response to C14MC may be related to the downregulation of *AKT* and *c-MYC* combined with the simultaneous upregulation of *p16* and *p27*.

### 3.9 C14MC activation inhibits EMT signaling

C14MC activation inhibited both migration and invasion. EMT pathway activation is key for the migration and invasion of CC cells. Hence, we analyzed the effect of C14MC on EMT signaling. C14MC-activated cells showed significant upregulation of *CDH1* (epithelial marker) and downregulation of mesenchymal markers such as *VIM* (Fig. 5E). In addition, we noted downregulation of *CCNE1* (Fig. 5E). These data suggested that C14MC is an inhibitor of the EMT pathway.

### 3.10 C14MC activation induces ROS, intracellular Ca^2+^, and lipid peroxidation

We investigated the changes in intracellular Ca^2+^ and ROS levels in response to C14MC activation by staining with Fluo3-AM and DCFDA, followed by confocal microscopy. The data revealed significant increases in ROS levels (Fig. 5F,G), intracellular Ca^2+^ levels (Fig. 5H,I), and lipid peroxidation rates (Fig. 5J) in C14MC-activated cells compared with scrambled vector-transfected control CaSki cells.

### 3.11 C14MC activation may promote metabolic reprogramming

We investigated the changes in glucose uptake and lactate production in response to C14MC activation. C14MC activation significantly reduced glucose uptake in CaSki cells (Fig. 5K). Furthermore, lactate production was also considerably reduced in the presence of C14MC (Fig. 5L).

### 3.12 Identification of C14MC-modulated genes in CaSki cells

**Fig. 6:**
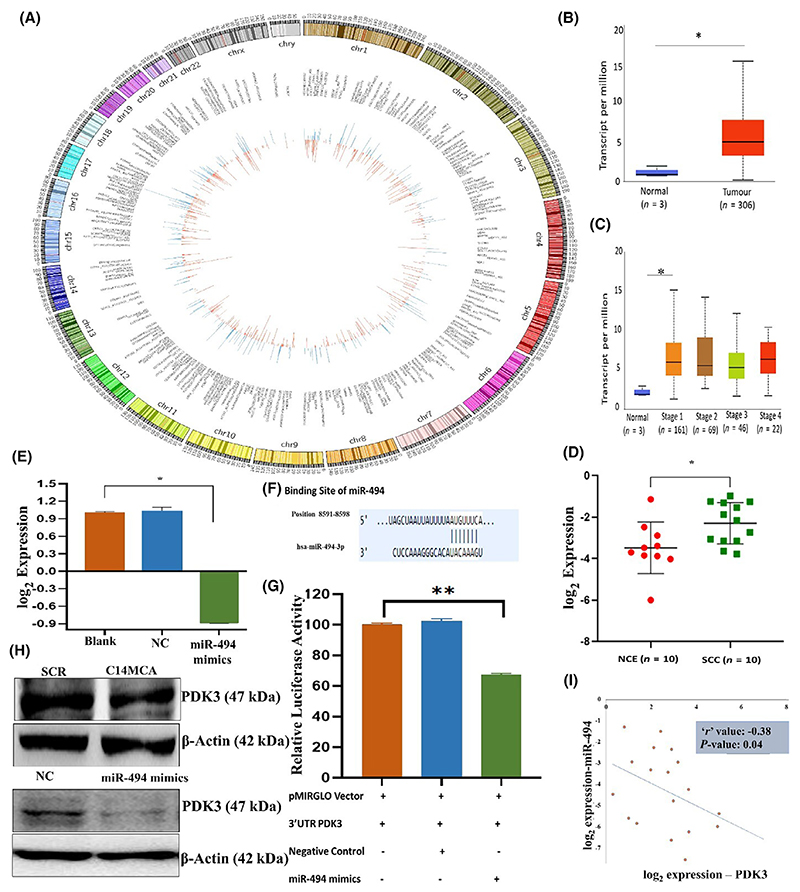
Transcriptomic analysis and validation identified *PDK3* as the target of C14MC. (A) Circos plot showing upregulated and downregulated genes upon C14MC activation in CaSki cells. Red and green represent the upregulated and downregulated genes, respectively. (B) Analysis of the TCGA-CESC dataset revealed that PDK3 was overexpressed in CC tissue samples compared with normal tissue sample. (C) Expression of the *PDK3* gene in different stages of CC in the TCGA-CESC cohort. (D) RT–PCR analysis confirmed the overexpression of *PDK3* in SCC (*n* = 10) compared with that in NCE (*n* = 10) samples. (E) Transfection of miR-494 mimics suppressed *PDK3* expression in SiHa cells. (F) miR-494 binds to the 3′UTR of *PDK3* according to TargetScan analysis. (G) Luciferase reporter assays confirmed that miR-494-3p directly targets *PDK3*. (H) Representative western blot image showing the downregulation of *PDK3* in C14MC-activated cells. (I) Pearson correlation analysis showing a negative correlation between miR-494 and *PDK3* expression in CC samples. Values in the graph represent mean ± SD of duplicates. Statistical significance was evaluated by unpaired Student’s *t*-test; *P < 0.05, (G) One-way ANOVA; **P < 0.01. Experiments are performed in duplicate and repeated three times.

Transcriptomic analysis revealed that C14MC activation modulated the expression of 1187 genes (482 upregulated and 705 downregulated; Fig. 6A, Table S5). The different classes of genes modulated in response to C14MC are protein-coding genes (Up: 396; Down: 593), lncRNA (Up: 51; Down: 65), processed pseudogenes (Up: 22; Down: 27), transcribed processed pseudogene (Up: 1; Down: 1), transcribed unitary pseudogene (Up: 1; Down: 4), transcribed unprocessed pseudogene (Up: 5; Down: 9), unprocessed pseudogene (Up: 6; Down: 4), misc RNA (Up: 0; Down: 1), and mitochondrial RNA (Up: 0; Down: 1). A search of the KEGG database revealed enrichment of cancer-related pathways, such as metabolic pathways, pathways in cancer, human papillomavirus infection, MAPK signaling, the cell cycle, miRNAs in cancer, PI3K-*AKT* signaling, calcium signaling, viral carcinogenesis, and proteoglycans in cancer.

### 3.13 *PDK3* is the direct target of C14MC in CC

The PDK family of proteins is reported to be an oncogene in several cancers, including CC [61]. Analysis of TCGA-CESC data revealed 2.86-fold upregulation of *PDK3* in CC samples compared to normal samples (Fig. 6B). The analysis of TCGA-CESC data showed that *PDK3* expression was significantly different between some of the histological subtypes of CC. Compared with those of other histological types, adenosquamous cell carcinoma samples showed significantly elevated expression of *PDK3* (Fig. 6C). Analysis of *PDK3* expression in our cohort of 10 normal and SCC samples revealed significant upregulation of PDK3 in tumor samples (Fig. 6D). miR-494 is a C14MC member predicted to target the *PDK3* gene. Transfection of miR-494 mimics and C14MC activation downregulated *PDK3* expression (Fig. 6E). *In silico* target prediction suggested that *PDK3* is targeted by 8 of the 42 members of C14MC (Table S6). We cloned the 3′UTR containing the miR-494-binding site in a luciferase reporter vector to confirm the C14MC-*PDK3* interaction. Cotransfection of these two constructs showed that miR-494 targets *PDK3* (Fig. 6F). Western blotting revealed significantly lower levels of *PDK3* expression in hsa-miR-494 mimic- and C14MC-activated cells than in negative control and scrambled control cells (Fig. 6H). In addition, there was a significant inverse correlation between C14MC and miR-494 (*r* = −0.38; Fig. 6I).

### 3.14 miR494 mimics inhibits proliferation, migration, and invasion of SiHa cells

**Fig. 7:**
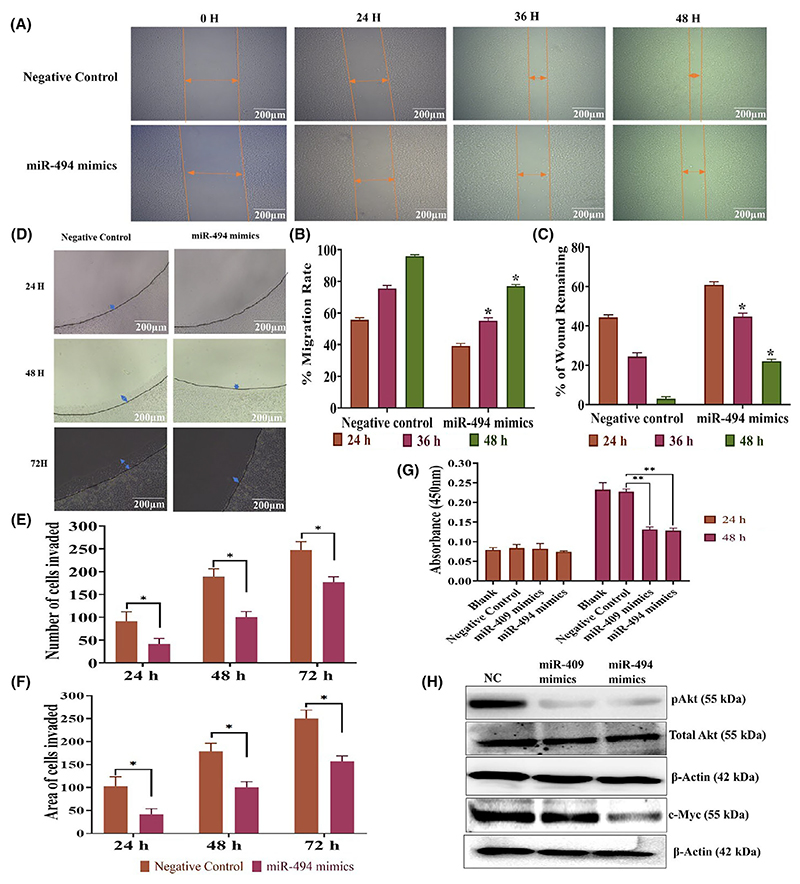
C14MC activation negatively regulates the migration and invasion of SiHa cells. (A) Representative images showing that miR-494 inhibits the migration of SiHa cells (Image Scale – 200 μm). (B, C) Bar graph showing the % migration rate and % of wound remaining in SiHa cells transfected with miR-494. Double arrow colored in orange indicates area of cells migrated. (D) Representative agarose spot assay showing that transfection with miR-494 significantly reduced the invasive ability of SiHa cells (Image Scale – 200 μm). (E, F) The number of cells invading the agarose spot and the depth of invasion, respectively. Double arrow colored in blue indicates area of cells invaded. (G) Bar graph showing a significant decrease in the proliferation rate of SiHa cells transiently transfected with miR-409 and miR-494 mimics. (H) Western blotting showed that transfection with miR-409 and miR-494 mimics inhibited the expression of the active forms of *AKT* and *c-MYC* in SiHa cells. Values in the graph represent mean ± SD of duplicates and statistical significance was performed by unpaired Student’s *t*-test, images were quantified using IMAGEJ software, *P < 0.05, (G) One-way ANOVA **P < 0.01. Experiments are performed in duplicate and repeated three times.

Transfection with miR-494 mimics significantly inhibited SiHa cell migration (Fig. 7A–C) and invasion (Fig. 7D–F) as evaluated by scratch wound assay and agarose spot invasion assay. Furthermore, we observed a significant reduction in cell proliferation rate in SiHa cells transfected with miR-494 mimics or miR-409 mimics (Fig. 7G). The western blot analysis showed that the miR-494 mimic reduced the active form of AKT and c-MYC without changing the total AKT level (Fig. 7H). However, transfection with miR-409 mimic showed the downregulation of only AKT (Fig. 7H).

## 4 Discussion

Cervical cancer is a lethal gynecological malignancy with increasing incidence and mortality rates in several countries. Despite the availability of screening techniques and treatment options, the 5-year overall survival rate of CC patients is still poor [53]. RNA-based therapeutics and biomarkers for cancer management have gained popularity in recent years [8]. C14MC is the second largest human miRNA cluster abnormally expressed in several malignancies and may act as a driver of tumorigenesis [17,25,62–64]. However, the role of the entire C14MC in CC pathology has not been determined. The present study is the first to investigate the regulation, biological function, and mechanism of the entire C14MC in CC by combining CC patient samples and *in vitro* models. CRISPR/Cas9-based C14MC activation had tumor-suppressive effects and noticeably (i) inhibited growth, proliferation, migration, and invasion; (ii) promoted actin cytoskeletal remodeling; reduced the number and length of filopodia; induced G2/M arrest; and increased senescence without significant changes in apoptosis; and (iii) decreased the expression of *c-MYC*, the active form of *AKT* and *CCNE*; decreased lactate production and glucose uptake; and increased reactive oxygen species (ROS), intracellular Ca^2+^, and lipid peroxidation. We showed that *PDK3* is the direct target of C14MC in CC. There was an inverse correlation between *PDK3* and C14MC expression in CC. The overexpression of *PDK3* due to the downregulation of C14MC may be involved in the development of CC. In summary, we identified C14MC as a methylation-driven tumor suppressor miRNA cluster and demonstrated that *PDK3* is a direct target of C14MC. Thus, targeting the C14MC-*PDK3* axis may be exploited for CC management.

The small RNA sequencing data from our study and miRNA expression data from TCGA-CESC showed significant downregulation of C14MC family members in CC tissues and cell lines. Thus, we propose that C14MC could be a tumor suppressor miRNA cluster in CC. The downregulation and tumor-suppressive functions of C14MC members have been reported in oligodendroglioma, rhabdomyosarcoma, ovarian cancer, prostate cancer, breast cancer, and gastrointestinal stromal cancer [23,65,66]. In contrast, the oncogenic role of C14MC members has also been reported in a few cancers [67,68]. These findings suggest that, depending on the tissue type and availability of the target genes, C14MC members may serve as tumor suppressors or oncogenes and may have diverse biological functions.

Promoter methylation controls the expression of miRNAs, and abnormal promoter methylation and miRNA expression have been detected in cancer cells [9,30,32]. Hypermethylation of upstream regions of C14MC correlated with decreased expression of C14MC miRNAs in a few cancers [22,69]. Additionally, hypomethylation of the C14MC locus was observed in lung adenocarcinoma [70], metastatic hepatoblastoma, acute promyelocytic leukemia, atherosclerosis, and Temple syndrome [71]. Hence, we investigated the impact of promoter methylation on C14MC expression, as we observed a global reduction in C14MC members in the CC. Our *in silico* analysis suggested that *MEF*2A may be a transcription factor for C14MC. The data generated from luciferase assays, bisulfite Sanger sequencing, and reactivation studies using 5-aza-2-deoxycytidine collectively showed that (i) C14MC is a hypermethylated and downregulated miRNA cluster and that (ii) the C14MC members are coexpressed in CC.

The measurement of miRNA expression can be considered a potential diagnostic and prognostic marker in CC, as miRNAs exhibit significant differences in expression in normal and tumor samples [72]. C14MC expression has significant potential for pathological classification, prognosis prediction (OS and DFS), and TNM staging in CC. Previous studies have reported that hsa-miR-381, hsa-miR-329, hsa-miR-376c, hsa-miR-379, and hsa-miR-494 are metastatic inhibitors of CC [54,56–58]. One limitation of our study is that the data used for pathological classification and prognosis prediction were from the TCGA-CESC dataset and require further investigation using independent datasets.

Abnormal cell cycle progression and cell division are highly affected pathways in CC and may promote CC progression by enhancing cell proliferation [73]. Pathway enrichment analysis revealed the involvement of cell cycle and cell division pathways, whose activation contributes to CC. For example, human papillomavirus infection and the PI3K-*AKT* signaling pathway are critical for HPV infection and cell cycle progression [74]. PPIN and its analysis identified ten hub genes (*CDK1, AURKA, BUB1, BIRC5, TTK, CCNB1, CEP55, MAD2L1, TOP2A*, and *KIF2C*). All ten hub genes of C14MC were reported to be overexpressed in CC, including in the TCGA-CESC cohort [75]. Interestingly, these genes have been shown to promote cell proliferation, cell cycle progression, aneuploidy, anti-apoptosis, malignant transformation, migration, and invasion. Thus, it is possible that the downregulation of C14MC may contribute to CC progression by inducing cell cycle arrest.

Abnormal expression of C14MC miRNAs contributes to carcinogenesis [23,65,66], but the functional relevance of the entire C14MC cluster in CC has not been determined. The CRISPRa approach and transfection of individual miRNAs from C14MC (miR-494, 409 mimic) demonstrated that coordinated overexpression of C14MC miRNAs inhibited the growth, proliferation, migration, and invasion of CC cells. Downregulation of C14MC has been shown to promote proliferation, migration, and invasion in ovarian cancer, melanoma [64], and SCC of the oral cavity [65]. Additionally, C14MC activation induced morphological changes, G2/M arrest, and senescence without inducing apoptosis. An earlier study showed that the overexpression of miR-485-5p, a member of the C14MC, blocked the proliferation of GBM cells [76]. C14MC-activated cells exhibited changes in cellular morphology and fewer filopodia, which may have contributed to the reduction in the migration rate of these cells.

Senescence can suppress tumorigenesis by inhibiting tumor cell proliferation. We noted strong G2/M arrest and *p16, p21*, and *p27* induction in response to C14MC activation in CaSki cells. Cell cycle arrest at G2/M phase has been associated with senescence [77]. Activation of *p16, p21*, and *p27* are known inducers of senescence and cell cycle arrest [78]. The senescence induction in response to C14MC may be due to an increase in the expression of *p16, p21*, and *p27*. Activation of C14MC-induced cell cycle arrest may reduce cell proliferation, growth, migration, and invasion by reducing the expression of cyclin E, *c-MYC*, and the active form of *AKT*. Pathway and functional enrichment analyses of C14MC targets in TCGA-CESC- and C14MC-activated CaSki cells suggested that the members of this cluster can target several well-established cancer-promoting pathways connected to CC, such as metabolic pathways involved in cancer, the cell cycle, *PI3K*-*AKT* signaling, human papillomavirus infection, and MAPK signaling. The inhibition of migration and invasion in the presence of C14MC could be attributed to EMT suppression due to *CDH1* upregulation and *VIM* downregulation. Taken together, the observed tumor-suppressive functions of C14MC could be attributed to the ability of C14MC to target multiple oncogenic pathways in CC.

Calcium ions (Ca^2+^) and ROS are key to biological processes that govern cell fate [79]. Hence, we analyzed the Ca^2+^ and ROS levels in control, C14MC-activated and miR-494 mimic-transfected cells. C14MC-activated and miR-494 mimic-transfected cells presented increased Ca^2+^ and ROS levels. In addition, we also observed significantly greater lipid peroxidation in C14MC-activated cells. Previous studies have shown that changes in Ca^2+^ and ROS levels and the lipid peroxidation rate inhibit cell proliferation and migration *in vitro* [80]. Furthermore, we observed a decrease in the glucose consumption rate and lactose production in C14MC, revealing its possible role in metabolic reprogramming in CC. Thus, increases in intracellular Ca^2+^ and ROS levels may have contributed to increased lipid peroxidation and the induction of senescence.

*PDK3*, located at Xp22.11, encodes a 46.93 kDa pyruvate dehydrogenase kinase protein and is a key enzyme that controls glucose metabolism by connecting glycolysis with the tricarboxylic acid (TCA) cycle [81]. *PDK3* is upregulated in colon [82], gastric [83], and prostate cancer [84]. Overexpression of *PDK3* is associated with poor prognosis in a few cancers [85]. The data of C14MC-activated cells, TCGA-CESC cohort and our cohort showed an inverse correlation between C14MC and *PDK3* expression. Target prediction analysis suggested that 8 miRNAs of C14MC can target *PDK3*. Our study and TCGA-CESC data analysis suggested significant overexpression of *PDK3* in CC. Hence, we propose that *PDK3* might act as an oncogene in CC. Among the three C14MC family members validated by qRT–PCR, only miR-494 had (i) the highest negative Pearson correlation coefficient compared to that of miR-376c and miR-495 in clinical samples and (ii) the highest cumulative score and repression ratio from TargetScan against *PDK3*. Furthermore, *PDK3* has emerged as a key regulator of metabolism and is upregulated in many cancers [82,86]. However, the role of *PDK3* has not been extensively investigated in CC. In addition, there are no previous reports on the miR-494-3p-*PDK3* axis in CC. Hence, miR-494 was further chosen for target validation. We showed that C14MC-activated and miR-494 mimic-transfected cells exhibited significant downregulation of *PDK3*. These data collectively suggest that the C14MC-*PDK3* axis may play an important role in CC. Targeting *PDK3* via C14MC could be employed in CC management.

## 5 Conclusion

We showed that C14MC is a hypermethylated and downregulated miRNA cluster in CC. Measuring the expression of C14MC may be useful for diagnostic and prognostic applications. Downregulation of C14MC correlated with poor prognosis and may promote metastasis. We showed that the activation of C14MC inhibited key biological properties of CC, including growth, proliferation, migration, and invasion, possibly via G2/M arrest. C14MC is a strong inducer of CaSki cell senescence. *In silico* and transcriptomic analyses showed that C14MC might target key pathways, such as the cell cycle pathway, that are considered CC drivers. C14MC-induced senescence could be due to enhanced expression of *p16, p21*, and *p27*. The downregulation of active *AKT, c-MYC*, and *CCNE* and the upregulation of *p16, p21*, and *p27* may reduce cell proliferation, growth, migration, and invasion, respectively. We showed that C14MC miRNA activation increases ROS, Ca^2+^, and lipid peroxidation rates and inhibits EMT. For the first time, we demonstrated that C14MC miRNAs target *PDK3*, a highly overexpressed gene in CC. However, additional detailed functional and mechanistic studies are needed to determine the C14MC–*PDK3* interaction and its role in CC. Overall, we propose the use of C14MC against CC.

## Supplementary Material

Supplementary figures

Supplementary tables

Supplementary figures and tables Legends

## Data Availability

The manuscript and its supporting files encompass all data generated or analyzed in this study. The data that support the findings of this study are available from the corresponding author upon reasonable request.

## Abbreviations

ACTB: Beta Actin
AKT: AKT serine/threonine kinase
AURKA: Aurora kinase A
BIRC5: baculoviral IAP repeat containing 5
BMI1: B cell-specific Moloney murine leukemia virus integration site 1
BP: Base Pair
BUB1: BUB1 mitotic checkpoint serine/threonine kinase
C14MC: Chromosome 14 miRNA Cluster
CC: Cervical Cancer
CCNB1: Cylin B1
CCNE1: Cyclin E1
CDH1: Cadherin 1
CDH2: Cadherin 2
CDK1: cyclin-dependent kinase 1
CDLUE1: Deleted in lymphocytic leukemia 1
CEP55: Centrosomal Protein 1
CRISPRa: Clustered Regulatory Interspaced Palindrome Repeats activation
CRKL: CRK Like Proto-Oncogene
DCFDA: Dichlorodihydrofluorescein diacetate
DFS: Disease Free Survival
DIO3: iodothyronine deiodinase 3
DLK1: Delta Like non-canonical Notch ligand 1
DMEM: Dulbecco’s Modified Eagle Medium
DNA: Deoxy Ribonucleic Acid
EDTA: Ethylenediaminetetraacetic acid
EGF: Epidermal Growth Factor
EMT: Epithelial-Mesenchymal Transition
FBS: Foetal Bovine Serum
FC: Fold Change
FGF7: Fibroblast Growth Factor 7
FP: Forward Primer
GBM: Glioblastoma
GO: Gene Ontology
H3K27ac: histone 3 lysine 27 acetylation
HCL: Hydrochloric Acid
HOXA13: Homeobox A13
HPV: Human Papillomavirus
KIF2C: Kinesin Family member 2C
MACC1: Metastasis-Associated in Colon Cancer 1
MAD2L1: Mitotic Arrest Deficient 2 Like 1
MAPK1: Mitogen-Activated Protein Kinase 1
MEF2: Myocyte Enhancer Factor 2
MEG3: Maternally Expressed 3
MEG8: Maternally expressed 8
miRNA: MicroRNA
MTF2: Metal Response Element Binding Transcription Factor 2
MYC: MYC proto-oncogene
NaCl: Sodium Chloride
NCE: Normal Cervical Epithelium
OS: Overall Survival
PDK3: Pyruvate Dehydrogenase Kinase-3
PI3K: Phosphatidylinositol 3-Kinase
PIPES: Piperazinediethanesulfonic Acid
PPIN: Protein–Protein Interaction Network
qRT-PCR: Quantitative real-time reverse-transcription PCR
RNA: Ribonucleic Acid
RNU6B: U6 Small Nuclear RNA
ROS: Reactive Oxygen Species
RP: Reverse Primer
RPMI: Roswell Park Memorial Institute
RTL1: Retrotransposon Gag like 1
SAM: Synergistic Activator Mediator
SCC: Squamous Cell Carcinoma
SDS/PAGE: Sodium Dodecyl Sulfate–Polyacrylamide Gel Electrophoresis
SGK3: Serum/Glucocorticoid regulated Kinase family member 3
sgRNA: single-guide RNA
SNAIL1: Snail Family Transcriptional Repressor 1
STAT3: Signal transducer and activator of transcription 3
STK17A: serine/threonine kinase 17a
SV40: Simion Virus 40
TACCO: Transcriptome Alterations in CanCer Omnibus
TBA: ThioBarbituric Acid
TBST: Tris-buffered saline with Tween20
TCGA: The Cancer Genome Atlas
TCGA-CESC: The Cancer Genome Atlas Cervical Squamous Cell Carcinoma and Endocervical Adenocarcinoma
TNM: Tumor, Node, Metastasis
TOP2A: DNA topoisomerase II alpha
TTK: TTK protein kinase
UTR: Untranslated Region
VIM: Vimentin
ZEB2: Zinc Finger E-Box Binding Homeobox 2
