# Supplementary material for: Regulation and tumor-suppressive function of the miR-379/miR-656 (C14MC) cluster in cervical cancer
Source: Mol Oncol. Author manuscript; Available in PMC 2024 Jun 10. (PMC11161731; doi:10.1002/1878-0261.13611)
Supplement: Supplementary figures [file EMS195193-supplement-Supplementary_figures.pdf]

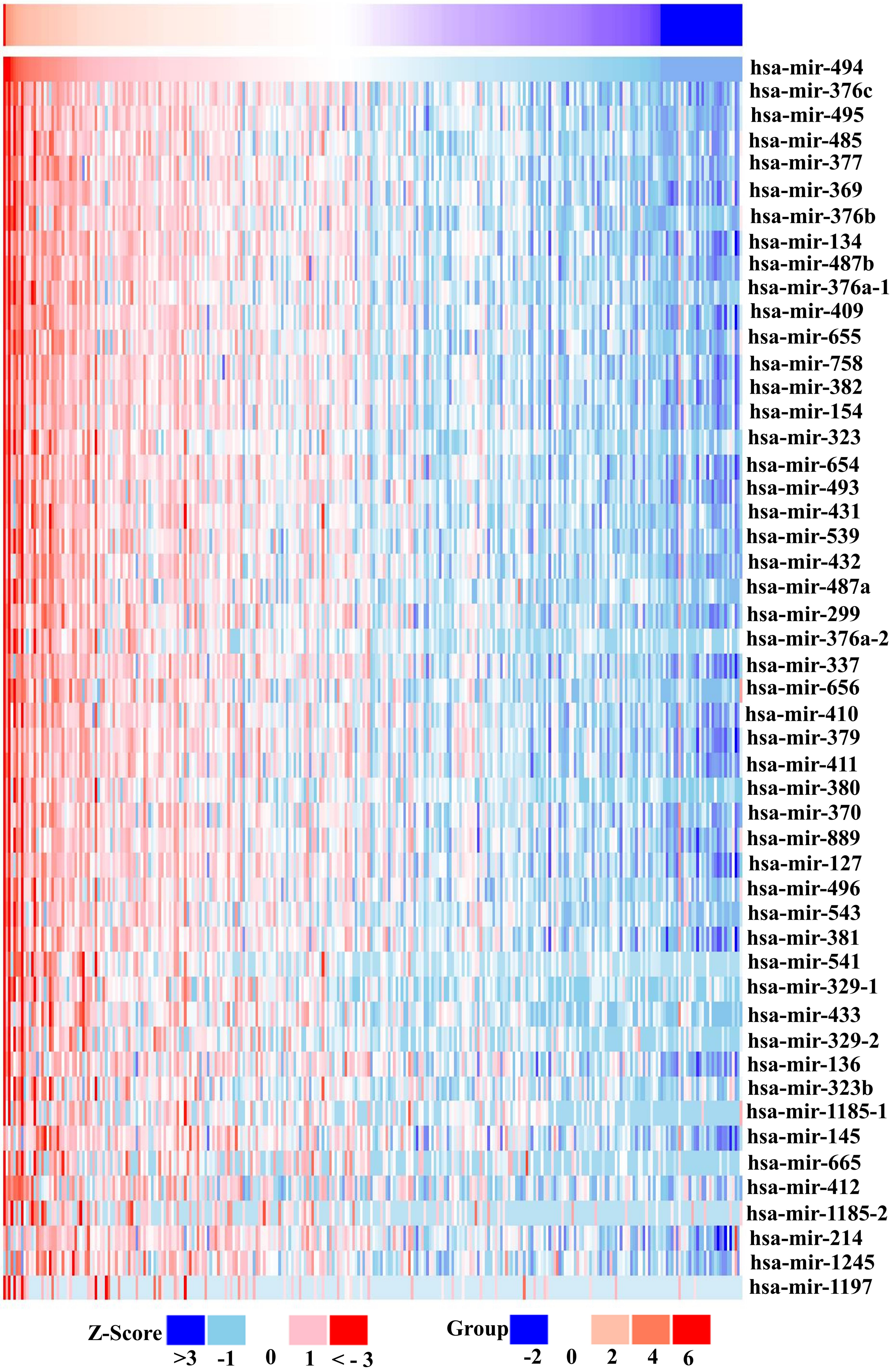
hsa-mir-494
hsa-mir-376c
hsa-mir-495
hsa-mir-485
hsa-mir-377
hsa-mir-369
hsa-mir-376b
hsa-mir-134
hsa-mir-487b
hsa-mir-376a-1
hsa-mir-409
hsa-mir-655
hsa-mir-758
hsa-mir-382
hsa-mir-154
hsa-mir-323
hsa-mir-654
hsa-mir-493
hsa-mir-431
hsa-mir-539
hsa-mir-432
hsa-mir-487a
hsa-mir-299
hsa-mir-376a-2
hsa-mir-337
hsa-mir-656
hsa-mir-410
hsa-mir-379
hsa-mir-411
hsa-mir-380
hsa-mir-370
hsa-mir-889
hsa-mir-127
hsa-mir-496
hsa-mir-543
hsa-mir-381
hsa-mir-541
hsa-mir-329-1
hsa-mir-433
hsa-mir-329-2
hsa-mir-136
hsa-mir-323b
hsa-mir-1185-1
hsa-mir-145
hsa-mir-665
hsa-mir-412
hsa-mir-1185-2
hsa-mir-214
hsa-mir-1245
hsa-mir-1197
Z-Score
>3
-1
0
1
< - 3
Group
-2
0
2
4
6

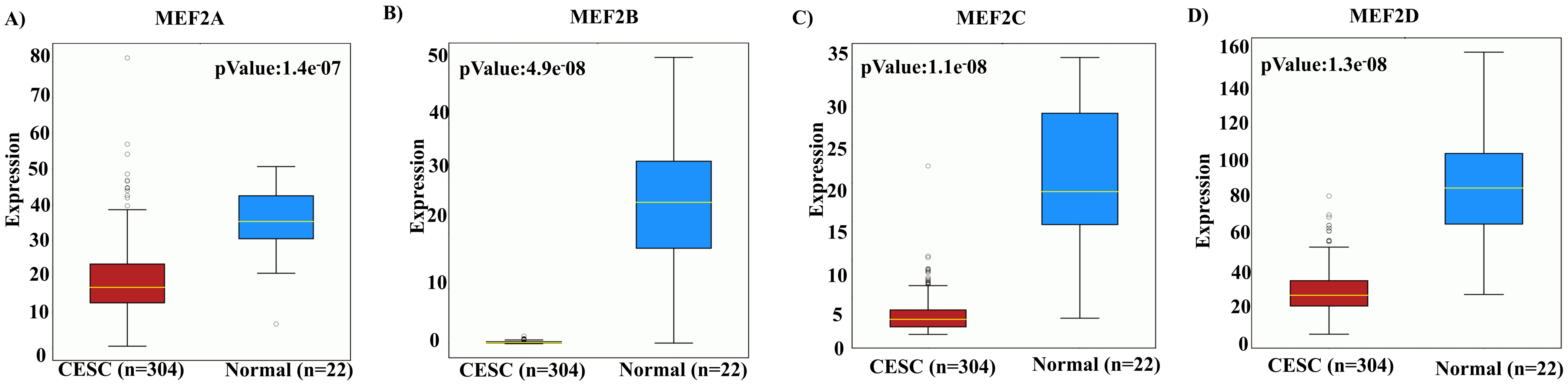

E) MEF2 Binding site prediction in promoter sequence

CCAACGTCCCACGTCTCTATCTCCCCAACAGTGCGCCTGTTTATGAAAAAACGAGCCCCCCACACGCCGTCCCAAGGCTCGGCGCCTCTAGTGACCTGACGGTC
AATGTTCACCTCCCTAGTCATTAGC**TGTGGACGTAGAAATAGCCTTT**CTCCCTTCTTTTCAGCCCTGGAATCTCCCGTCTGCTTTACAGTCACCTTGTGGGATCG
TTGGGCATGGGGGGCTCCCTCTGACGCCGCTTAAACCCCCCAAAGAAGTGCGGGGGAGCGATTCTGTTGATGCCCTCGGCGGGGCCTCTCTGCTGTCCTCTTTG
GGGCCCCTTGCTCATCCTCACCTGCTTTCTGTCACCGAGCACTCCATCCTTGTCCTCCTGGCTGCCACCCACCTGTCACCCTAAGATAGATCCTCGGTCCCTTTCA
ACAGCATTTTGACCTTTGCGAGAGGACATTTGATTGACGGCCCTGCACGCTCTGCCACTCAGAGCCGGGCAGAGCTGCCGAGGGCTCCCACCTGTTAGGGATTA
ACTCCCATGTGCCAGCTCCGGAGCCGAGGCCGCGGCAGGGCTCGGCGCAACATGTGTCGCTGCCTGTTTTTCGGGGCGCTGGGGCTCTTTCAAAGGGCATGTGT
GTGGGGATCACCCCAACTTTGAGGGGTAGCTCAGGCATCTCCACATGGGACACAGTCCGCC**CTCTAAAAGTACTTTC**CTTAGAGGGCACGGCTCCTGTCTGGAT
TCTTATTACAACCCTGGTGTGTGTGGTGGGGGGTACCCCTGGTTAAGGTGGAGGAAGCGGACCCAGTGGAAGAGGAAGGGGCATAGCGGGTTCCGCGAACATG
AGTTGTAAGCGGCAGAGCCCGGGACTCCACAGCCAGGGTTTTCTGACTCGGAGACGCGGAGCTCTGTCTCCCATGTCAAAGAGAACCAGTCAGAAACGCACG
GGTGGGCTGGGAGGGGGTGACAGCCTCTGGCTTACATCTGGGAACCAGTTATGTCGCCCGGGCATTTTATTTTCCTGAACAATAAGAGAAAGCATGATTTTCTTT
CGCTAAGCCCGCATCCTCCGATGGATGTTCCGAAACCGCCAGGTGTGGGATCTGCGCCCCGACAGCCCCACCTTGGCCATCGGCCGCCTGAGGACGGCCAGCCA
TCGGGGCGATTCCCCCCACACATTGTGCCTGAATTCACCCTGCCTGGCGAGCCGGGCTCACGCAGGGAAAAAGCACCCGCGACCACAGGGTGTTGGTCATGGC
GGCCAGGGGACTGCGGCAGAATTTTTTCCTCCCTTCTTTGCTGCAATCTGGGTGCGGCTAGAGCAATTTGTCATAGAATCTGGGGGGCTCATTTTTCCGGCCAATC
ACTTTTAGAGAAATGAGCGCATTGCAGCAGAATGCGCTGACGTCAGAGACCACCCCTTCTGCGCCTCCATATAAACC

A)

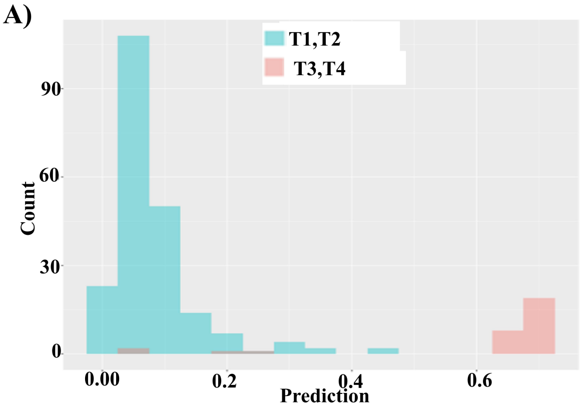

Prediction/ Observation	T1,T2	T3,T4	SubTotal
T1,T2	211	4	215
T3,T4	0	27	27
SubTotal	211	31	

Sensitivity: 1 **Specificity: 0.87**

B)

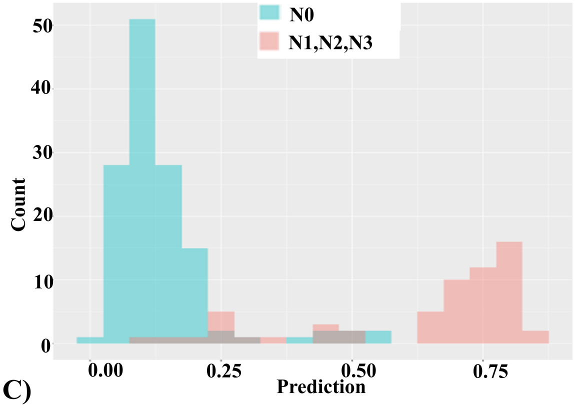

Prediction/ Observation	N0	N1,N2,N3	SubTotal
N0	130	14	144
N1,N2,N3	3	46	49
SubTotal	133	60	

Sensitivity: 0.98 **Specificity: 0.77**

C)

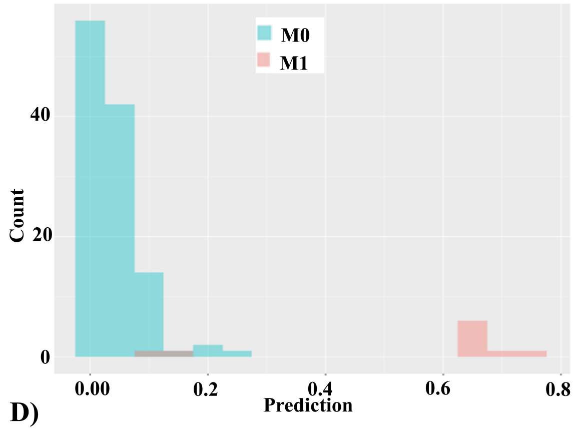

Prediction/ Observation	M0	M1	SubTotal
M0	116	2	118
M1	0	8	8
SubTotal	116	10	

Sensitivity: 1 **Specificity: 0.8**

D)

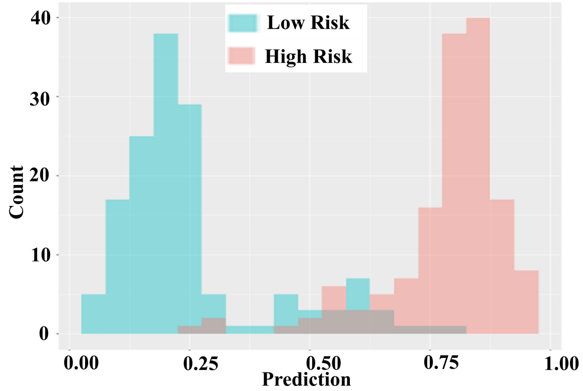

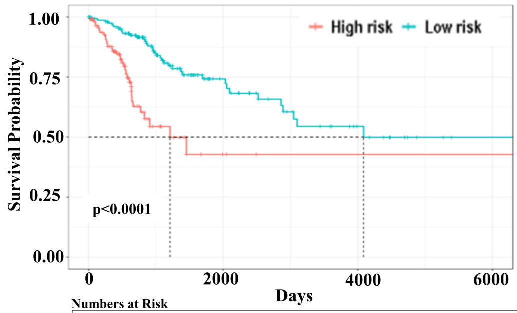

Numbers at Risk

High Risk	129	3	1	1
Low Risk	156	37	12	1

E)

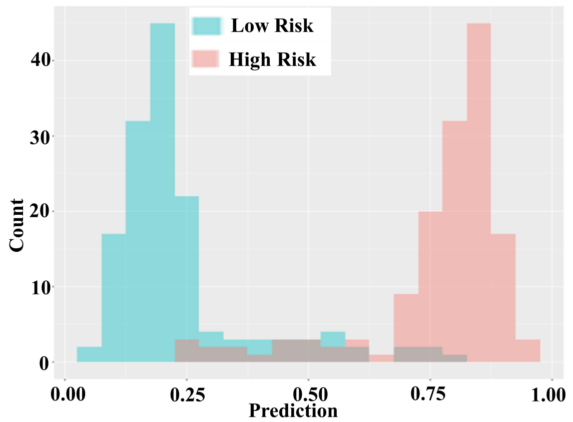

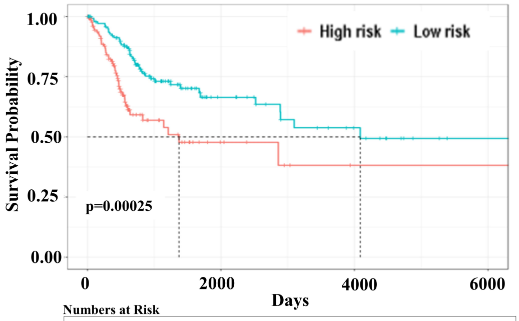

Numbers at Risk

High Risk	140	7	1	1
Low Risk	145	28	12	1

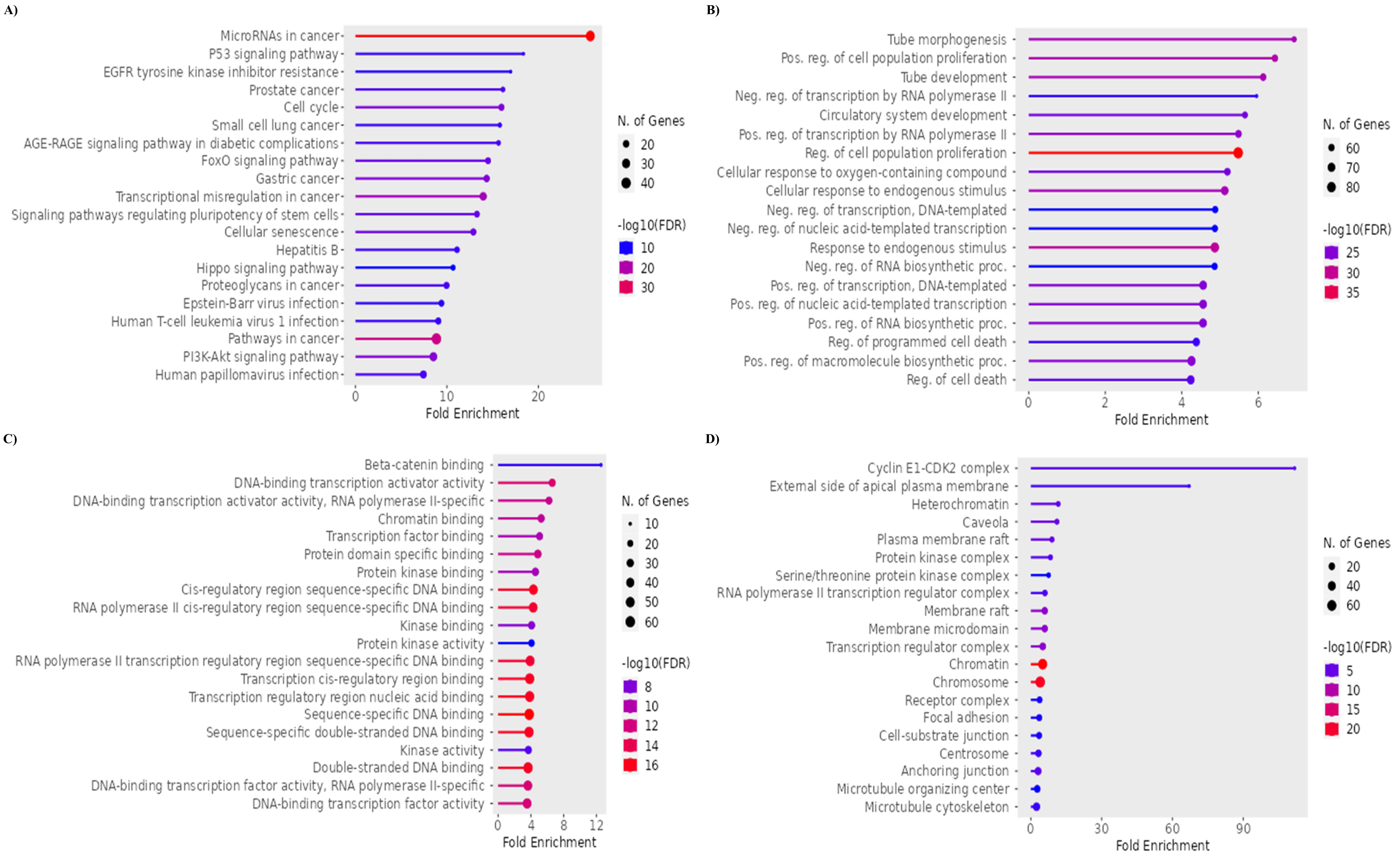
A)
MicroRNAs in cancer
P53 signaling pathway
EGFR tyrosine kinase inhibitor resistance
Prostate cancer
Cell cycle
Small cell lung cancer
AGE-RAGE signaling pathway in diabetic complications
FoxO signaling pathway
Gastric cancer
Transcriptional misregulation in cancer
Signaling pathways regulating pluripotency of stem cells
Cellular senescence
Hepatitis B
Hippo signaling pathway
Proteoglycans in cancer
Epstein-Barr virus infection
Human T-cell leukemia virus 1 infection
Pathways in cancer
PI3K-Akt signaling pathway
Human papillomavirus infection
0
10
20
Fold Enrichment
N. of Genes
20
30
40
-log10(FDR)
10
20
30
B)
Tube morphogenesis
Pos. reg. of cell population proliferation
Tube development
Neg. reg. of transcription by RNA polymerase II
Circulatory system development
Pos. reg. of transcription by RNA polymerase II
Reg. of cell population proliferation
Cellular response to oxygen-containing compound
Cellular response to endogenous stimulus
Neg. reg. of transcription, DNA-templated
Neg. reg. of nucleic acid-templated transcription
Response to endogenous stimulus
Neg. reg. of RNA biosynthetic proc.
Pos. reg. of transcription, DNA-templated
Pos. reg. of nucleic acid-templated transcription
Pos. reg. of RNA biosynthetic proc.
Reg. of programmed cell death
Pos. reg. of macromolecule biosynthetic proc.
Reg. of cell death
0
2
4
6
Fold Enrichment
N. of Genes
60
70
80
-log10(FDR)
25
30
35
C)
Beta-catenin binding
DNA-binding transcription activator activity
DNA-binding transcription activator activity, RNA polymerase II-specific
Chromatin binding
Transcription factor binding
Protein domain specific binding
Protein kinase binding
Cis-regulatory region sequence-specific DNA binding
RNA polymerase II cis-regulatory region sequence-specific DNA binding
Kinase binding
Protein kinase activity
RNA polymerase II transcription regulatory region sequence-specific DNA binding
Transcription cis-regulatory region binding
Transcription regulatory region nucleic acid binding
Sequence-specific DNA binding
Sequence-specific double-stranded DNA binding
Kinase activity
Double-stranded DNA binding
DNA-binding transcription factor activity, RNA polymerase II-specific
DNA-binding transcription factor activity
0
4
8
12
Fold Enrichment
N. of Genes
10
20
30
40
50
60
-log10(FDR)
8
10
12
14
16
D)
Cyclin E1-CDK2 complex
External side of apical plasma membrane
Heterochromatin
Caveola
Plasma membrane raft
Protein kinase complex
Serine/threonine protein kinase complex
RNA polymerase II transcription regulator complex
Membrane raft
Membrane microdomain
Transcription regulator complex
Chromatin
Chromosome
Receptor complex
Focal adhesion
Cell-substrate junction
Centrosome
Anchoring junction
Microtubule organizing center
Microtubule cytoskeleton
0
30
60
90
Fold Enrichment
N. of Genes
20
40
60
-log10(FDR)
5
10
15
20

A)

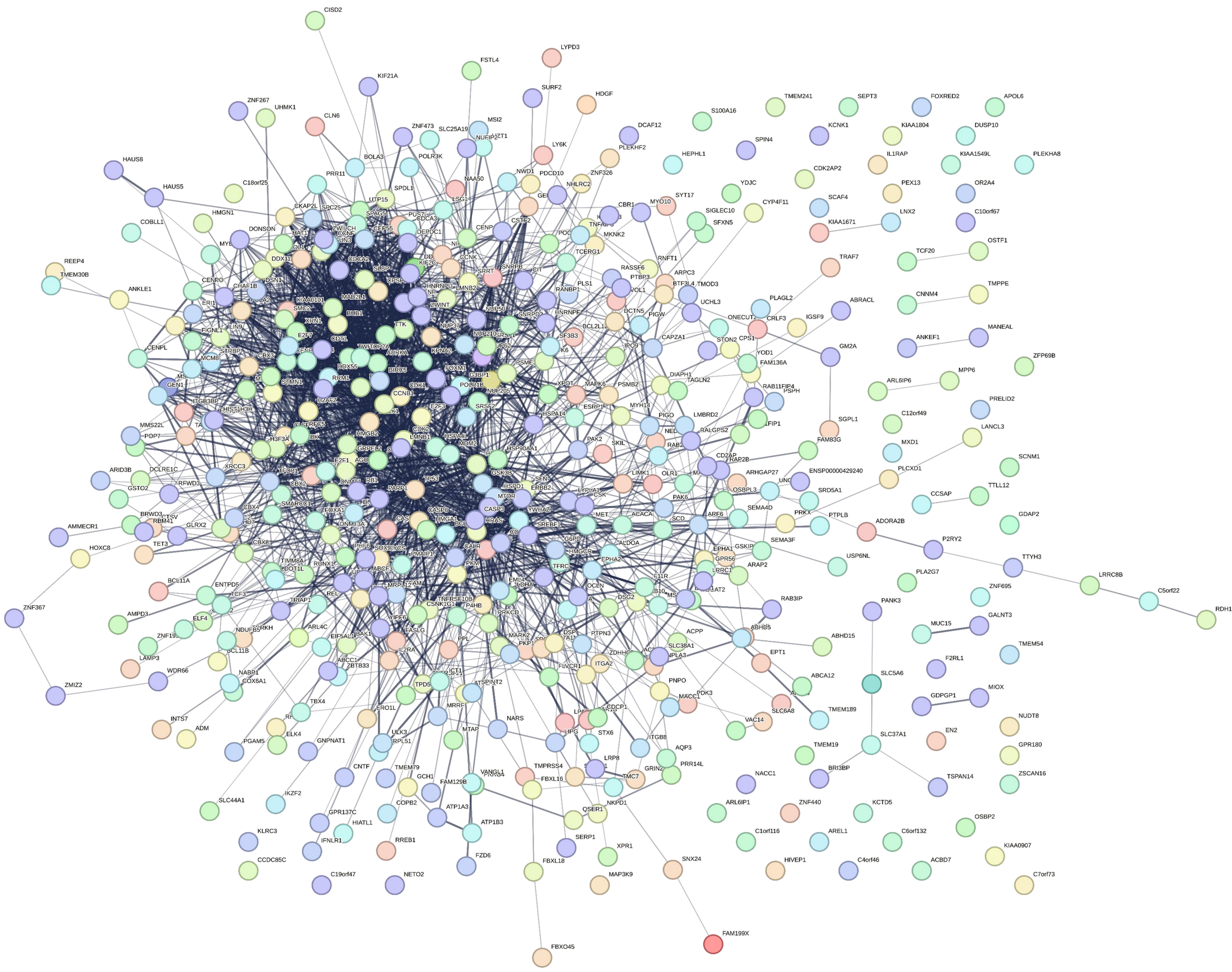

B)

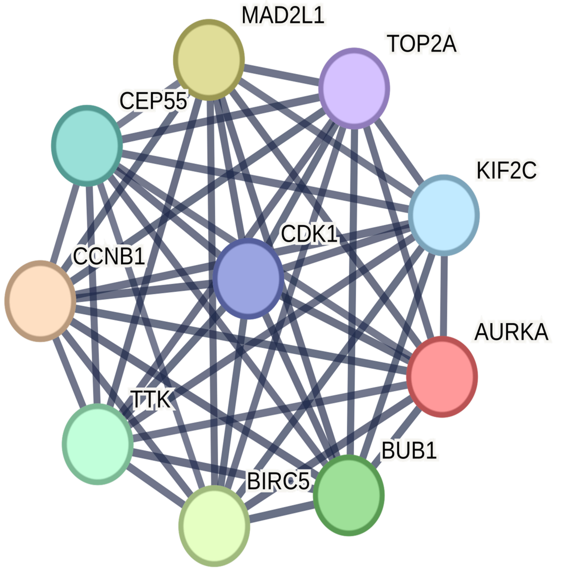

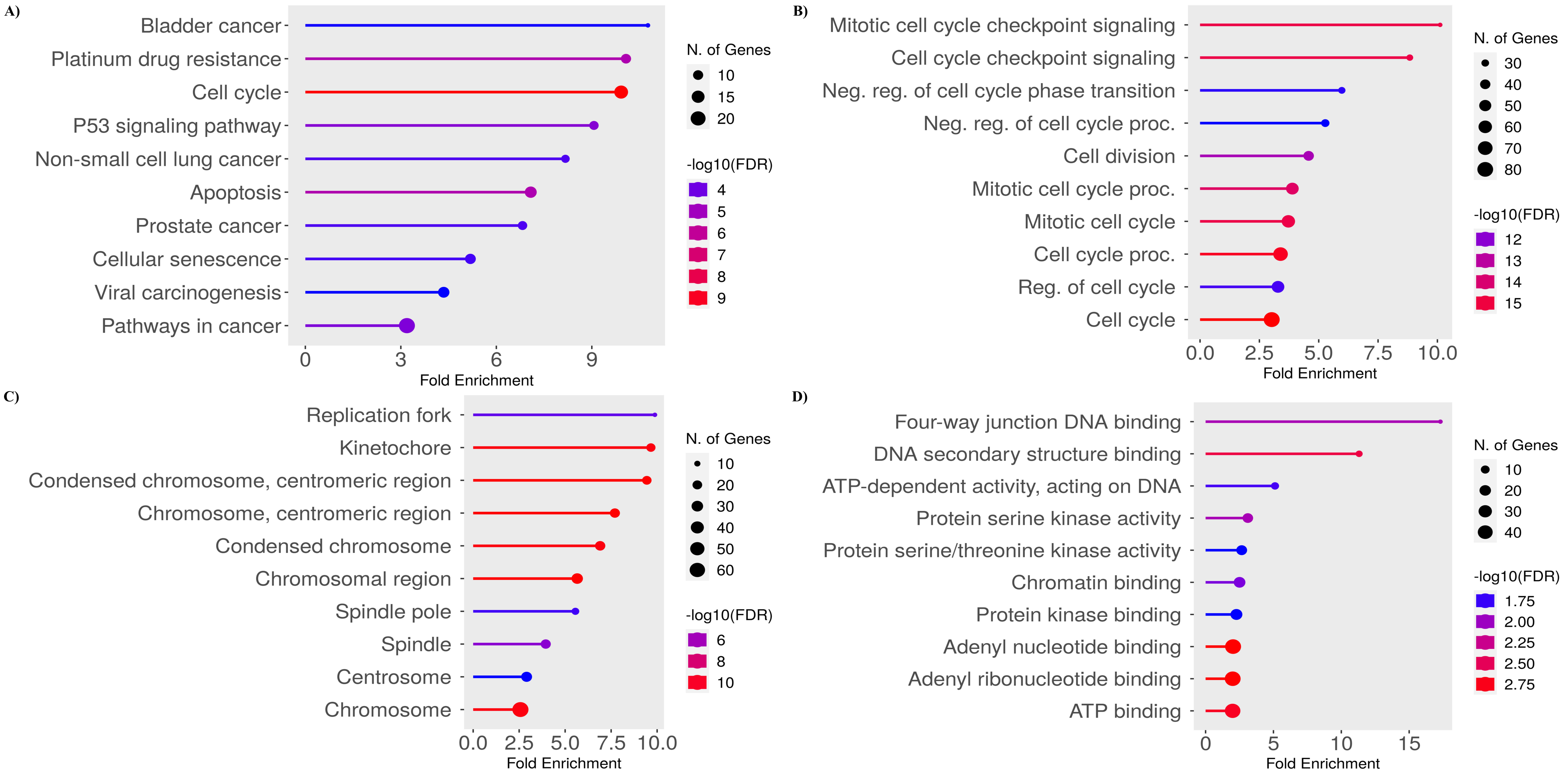
A)
Bladder cancer
Platinum drug resistance
Cell cycle
P53 signaling pathway
Non-small cell lung cancer
Apoptosis
Prostate cancer
Cellular senescence
Viral carcinogenesis
Pathways in cancer
0
3
6
9
Fold Enrichment
N. of Genes
10
15
20
-log10(FDR)
4
5
6
7
8
9
B)
Mitotic cell cycle checkpoint signaling
Cell cycle checkpoint signaling
Neg. reg. of cell cycle phase transition
Neg. reg. of cell cycle proc.
Cell division
Mitotic cell cycle proc.
Mitotic cell cycle
Cell cycle proc.
Reg. of cell cycle
Cell cycle
0.0
2.5
5.0
7.5
10.0
Fold Enrichment
N. of Genes
30
40
50
60
70
80
-log10(FDR)
12
13
14
15
C)
Replication fork
Kinetochore
Condensed chromosome, centromeric region
Chromosome, centromeric region
Condensed chromosome
Chromosomal region
Spindle pole
Spindle
Centrosome
Chromosome
0.0
2.5
5.0
7.5
10.0
Fold Enrichment
N. of Genes
10
20
30
40
50
60
-log10(FDR)
6
8
10
D)
Four-way junction DNA binding
DNA secondary structure binding
ATP-dependent activity, acting on DNA
Protein serine kinase activity
Protein serine/threonine kinase activity
Chromatin binding
Protein kinase binding
Adenyl nucleotide binding
Adenyl ribonucleotide binding
ATP binding
0
5
10
15
Fold Enrichment
N. of Genes
10
20
30
40
-log10(FDR)
1.75
2.00
2.25
2.50
2.75
