# Supplementary material for: Regulation and tumor-suppressive function of the miR-379/miR-656 (C14MC) cluster in cervical cancer
Source: Mol Oncol. Author manuscript; Available in PMC 2024 Jun 10. (PMC11161731; doi:10.1002/1878-0261.13611)
Supplement: Supplementary tables [file EMS195193-supplement-Supplementary_tables.pdf]

Supplementary Table 1 : Cell Line authentication Using GenePrint-10 System by STR Profiling

Cell lines with ≥90% match are considered to be related; derived from a common ancestry. Cell lines with between a 55% to 80% match require further analysis for authentication of relatedness.

Cell line	Loci	Allele (Repeat)	% Match	ATCC No.	Designation
SiHa	TH01	6,9	100	HTB - 35	SiHa Cervical carcinoma Human
	D21S11	29,30			
	D5S818	11,12			
	D13S317	11,12			
	D7S820	10			
	D16S539	11,12			
	AMEL	X			
	v WA	17,19			
	TPOX	8,9			
	CSFIP0	11,12			
CaSki	TH01	6,9	100	CRL - 1550	CaSki Cervical Carcinoma Human
	D21S11	29,31			
	D5S818	9			
	D13S317	11			
	D7S820	10			
	D16S539	12			
	AMEL	X			
	v WA	14,17			
	TPOX	8			
	CSFIP0	12			
HeLa	TH01	7,9.3	93	CCL-2.2	HeLa S3Cervical Adenocarcinoma Human
	D21S11	33.2			
	D5S818	12			
	D13S317	13			
	D7S820	8,9			
	D16S539	12			
	AMEL	X			
	v WA	15,18			
	TPOX	8,9			
	CSFIP0	12,13			

Supplementary Table 2: Antibodies used for performing Western blotting.

S.No	Protein	Catalogue Number	Dilution	Source	Company
1	p-Akt, Total Akt	# 44-621G	1:3000	Rabbit	CST
2	c-Myc	# MA1-980	1:2500	Mouse	Thermo
3	CCNE	sc-377100	1:3000	Rabbit	SantaCruz
4	p16	sc-56330	1:2500	Rabbit	SantaCruz
5	p21	sc-397	1:2500	Mouse	SantaCruz
6	p27	sc-528	1:2500	Rabbit	SantaCruz
7	Actin	AC026	1:3000	Mouse	CST
8	CDH1	24E10	1:3000	Mouse	CST
9	VIM	MAB040Hu22	1:3000	Rabbit	Cloud Clone
10	SNAI1	MAK089Hu21	1:3000	Rabbit	Cloud Clone
11	CDH2	# 13116	1:3000	Mouse	CST
12	PDK3	A8028	1:3000	Rabbit	AbClonal

Supplementary Table 3: TCGA expression profile of C14MC Cluster in Cervical Cancer

MiRNA	miRNA.Accession	logFC	AveExpr	t score	P.Value	adj.P.Val	B statistics	Significance
hsa-miR-134-5p	MIMAT0000447	-1.8226	6.932	-2.40191	0.0169	0.0419	-3.7068	DOWN
hsa-miR-154-5p	MIMAT0000452	-1.98656	1.2173	-2.49848	0.013	0.034	-3.4791	DOWN
hsa-miR-299-5p	MIMAT0002890	-2.45916	0.9423	-2.97613	0.00315	0.0107	-2.2267	DOWN
hsa-miR-323a-3p	MIMAT0000755	-0.71767	0.3549	-0.7464	0.456	0.573	-6.2385	NS
hsa-miR-323b-3p	MIMAT0015050	0.81158	0.2221	0.90276	0.367	0.496	-6.1122	NS
hsa-miR-369-5p	MIMAT0001621	-1.97622	1.1576	-2.07368	0.0389	0.0806	-4.4161	DOWN
hsa-miR-369-3p	MIMAT0000721	-0.70502	2.1718	-0.89241	0.373	0.501	-6.1213	NS
hsa-miR-376c-3p	MIMAT0000720	-2.41263	1.9431	-2.7219	0.00685	0.0206	-2.9192	DOWN
hsa-miR-379-5p	MIMAT0000733	-2.53292	8.7236	-3.43432	0.000674	0.00289	-0.8323	DOWN
hsa-miR-381-3p	MIMAT0000736	-2.99554	4.9664	-4.51808	0.00000886	0.0000818	3.1857	DOWN
hsa-miR-382-5p	MIMAT0000737	-1.82546	3.0745	-2.64949	0.00847	0.0239	-3.1056	DOWN
hsa-miR-382-3p	MIMAT0022697	-1.77985	0.2087	-2.0405	0.0421	0.0852	-4.4821	DOWN
hsa-miR-494-3p	MIMAT0001638	-1.51728	2.5021	-2.08787	0.0376	0.0791	-4.3875	DOWN
hsa-miR-409-3p	MIMAT0001639	-0.83381	3.3599	-1.1912	0.234	0.342	-5.8169	NS
hsa-miR-410-3p	MIMAT0002171	-2.09407	2.6002	-2.50571	0.0127	0.0337	-3.4616	DOWN
hsa-miR-411-5p	MIMAT0003329	-2.28362	1.9085	-2.66935	0.008	0.023	-3.055	DOWN
hsa-miR-412-5p	MIMAT0026557	-1.30207	1.1659	-1.18244	0.238	0.344	-5.8271	NS
hsa-miR-485-3p	MIMAT0002176	-1.5547	1.0267	-1.85228	0.0649	0.125	-4.8373	NS
hsa-miR-487b-3p	MIMAT0003180	-2.1298	1.5078	-2.79707	0.00548	0.0173	-2.7205	DOWN
hsa-miR-495-3p	MIMAT0002817	-2.35498	2.0078	-3.06138	0.00239	0.00835	-1.9814	DOWN
hsa-miR-539-5p	MIMAT0003163	-1.95604	0.7676	-2.23756	0.026	0.0593	-4.0745	DOWN
hsa-miR-654-3p	MIMAT0004814	-2.10979	3.71	-2.61859	0.00926	0.0255	-3.1838	DOWN
hsa-miR-654-5p	MIMAT0003330	-1.81666	-0.2381	-2.16848	0.0309	0.0683	-4.2216	DOWN
hsa-miR-655-3p	MIMAT0003331	-1.63784	0.6927	-2.05363	0.0408	0.0833	-4.4561	DOWN
hsa-miR-758-5p	MIMAT0022929	-2.3908	1.5907	-2.64119	0.00868	0.0243	-3.1267	DOWN
hsa-miR-758-3p	MIMAT0003879	-1.97887	2.2003	-2.24566	0.0254	0.0584	-4.057	NS
hsa-miR-889-3p	MIMAT0004921	-1.45487	3.3089	-2.07263	0.039	0.0806	-4.4182	D0WN

Supplementary Table 4: C14MC members expression, roc curve and survival analysis performed in cervical cancer using miRNOME

S.No	miRNA	Expression (p-value)	AUC		Survival Analysis	
					HR	p-value
1	hsa-miR-379-3p	0.02	0.89	(95%CI:0.75-1.00)	1.47(0.93-2.35)	0.11
2	hsa-miR-411-3p	0.01	0.96	(95%CI:0.92-1.00)	1.16(0.73-1.84)	0.54
3	hsa-miR-299-3p	0.01	0.94	(95%CI:0.88-0.99)	1.01(0.63-1.61)	0.97
4	hsa-miR-380-3p	0.28	0.66	(95%CI:0.29-1.00)	0.78(0.48-1.25)	0.31
5	hsa-miR-1197-3p	Not Available				
6	hsa-miR-323a-3p	0.29	0.68	(95%CI:0.52-0.83)	1.23(0.77-1.96)	0.38
7	hsa-miR-758-3p	0.03	0.87	(95%CI:0.69-1.00)	1.04(0.65-1.66)	0.87
8	hsa-miR-329-3p	0.04	0.85	(95%CI:0.70-0.99)	1.38(0.87-2.20)	0.18
9	hsa-miR-494-3p	0.59	0.41	(95%CI:0.05-0.77)	1.48(0.93-2.36)	0.1
10	hsa-miR-1193-3p	Not Available				
11	hsa-miR-543-3p	0.05	0.82	(95%CI:0.56-1.00)	1.09(0.68-1.73)	0.72
12	hsa-miR-495-3p	0.01	0.96	(95%CI:0.92-1.00)	1.04(0.65-1.66)	0.86
13	hsa-miR-376c-3p	Not Available				
14	hsa-miR-376a-3p	0.08	0.79	(95%CI:0.51-1.00)	1.45(0.91-2.31)	0.12
15	hsa-miR-300-3p	Not Available				
16	hsa-miR-1185-1-3p	0.25	0.67	(95%CI:0.31-1.00)	0.75(0.46-1.21)	0.25
17	hsa-miR-1185-2-3p	Not Available				
18	hsa-miR-381-3p	0.00	0.99	(95%CI:0.98-1.00)	0.86(0.54-1.36)	0.51
19	hsa-miR-487a-3p	0.37	0.65	(95%CI:0.18-1.00)	1.27(0.80-2.02)	0.32
20	hsa-miR-487b-3p	0.01	0.95	(95%CI:0.90-1.00)	1.03(0.65-1.64)	0.9
21	hsa-miR-539-3p	0.23	0.7	(95%CI:0.44-0.96)	1.79(1.12-2.86)	0.015
22	hsa-miR-889-3p	0.06	0.81	(95%CI:0.54-1.00)	1.19(0.75-1.90)	0.47
23	hsa-miR-543-3p	0.05	0.82	(95%CI:0.56-1.00)	1.09(0.68-1.73)	0.72
24	hsa-miR-655-3p	0.03	0.87	(95%CI:0.74-1.00)	1.65(1.03-2.63)	0.036
25	hsa-miR-382-3p	0.02	0.9	(95%CI:0.74-1.00)	1.17(0.73-1.87)	0.51
26	hsa-miR-134-3p	0.13	0.75	(95%CI:0.57-0.93)	0.86(0.54-1.38)	0.54
27	hsa-miR-668-3p	Not Available				
28	hsa-miR-485-3p	0.06	0.82	(95%CI:0.55-1.00)	1.22(0.76-1.94)	0.41
29	hsa-miR-154-3p	0.04	0.84	(95%CI:0.71-0.98)	1.06(0.67-1.69)	0.8
30	hsa-miR-496-3p	0.43	0.63	(95%CI:0.28-0.99)	1.40(0.88-2.23)	0.16
31	hsa-miR-377-3p	0.04	0.84	(95%CI:0.59-1.00)	0.86(0.54-1.37)	0.52
32	hsa-miR-541-3p	0.10	0.77	(95%CI:0.54-1.00)	1.13(0.71-1.80)	0.61
33	hsa-miR-409-3p	0.30	0.67	(95%CI:0.16-1.00)	1.45(0.91-2.31)	0.12
34	hsa-miR-412-3p	0.19	0.72	(95%CI:0.33-1.00)	1.22(0.77-1.94)	0.4
35	hsa-miR-369-3p	0.29	0.68	(95%CI:0.23-1.00)	1.61(1.01-2.56)	0.046
36	hsa-miR-410-3p	0.02	0.9	(95%CI:0.81-0.99)	1.21(0.76-1.92)	0.43
37	hsa-miR-656-3p	0.03	0.87	(95%CI:0.78-0.96)	1.51(0.95-2.40)	0.085
38	hsa-miR-379-5p	0.01	0.97	(95%CI:0.94-1.00)	0.96(0.60-1.53)	0.87
39	hsa-miR-411-5p	0.01	0.95	(95%CI:0.90-1.00)	1.45(0.91-2.31)	0.12
40	hsa-miR-299-5p	Not Available				
41	hsa-miR-380-5p	0.07	0.81	(95%CI:0.57-1.00)	1.06(0.66-1.69)	0.81
42	hsa-miR-1197-5p	Not Available				
43	hsa-miR-323a-5p	Not Available				
44	hsa-miR-758-5p	0.01	0.93	(95%CI:0.86-1.00)	1.30(0.82-2.07)	0.27

45	hsa-miR-329-5p	Not Available				
46	hsa-miR-494-5p	0.06	0.72	(95%CI:0.41-1.00)	1.35(0.72-2.53)	0.3
47	hsa-miR-1193-5p	Not Available				
48	hsa-miR-543-5p	Not Available				
49	hsa-miR-495-5p	0.07	0.75	(95%CI:0.34-1.00)	0.54(0.33-0.88)	0.03
50	hsa-miR-376c-5p	Not Available				
51	hsa-miR-376a-5p	0.47	0.62	(95%CI:0.20-1.00)	0.90(0.56-1.43)	0.65
52	hsa-miR-300-5p	Not Available				
53	hsa-miR-1185-1-5p	Not Available				
54	hsa-miR-1185-2-5p	Not Available				
55	hsa-miR-381-5p	0.02	0.89	(95%CI:0.72-1.00)	0.86(0.54-1.37)	0.53
56	hsa-miR-487-5p	Not Available				
57	hsa-miR-539-5p	0.04	0.84	(95%CI:0.61-1.00)	1.05(0.66-1.66)	0.85
58	hsa-miR-889-5p	Not Available				
59	hsa-miR-543-5p	Not Available				
60	hsa-miR-655-5p	Not Available				
61	hsa-miR-382-5p	0.01	0.93	(95%CI:0.88-0.99)	1.45(0.91-2.30)	0.12
62	hsa-miR-134-5p	0.02	0.9	(0.78-1.00)	1.09(0.68-1.73)	0.72
63	hsa-miR-668-5p	Not Available				
64	hsa-miR-485-5p	0.01	0.92	(95%CI:0.84-1.00)	0.81(0.51-1.29)	0.37
65	hsa-miR-154-5p	0.02	0.9	(95%CI:0.8-1.00)	0.84(0.53-1.34)	0.47
66	hsa-miR-496-5p	Not Available				
67	hsa-miR-377-5p	0.01	0.95	(95%CI:0.87-1.00)	1.01(0.63-1.60)	0.98
68	hsa-miR-541-5p	Not Available				
69	hsa-miR-409-5p	0.03	0.87	(95%CI:0.73-1.00)	0.71(0.45-1.14)	0.16
70	hsa-miR-412-5p	Not Available				
71	hsa-miR-369-5p	0.02	0.89	(95%CI:0.78-1.00)	1.25(0.79-1.99)	0.35
72	hsa-miR-410-5p	0.14	0.7	(95%CI:0.34-1.00)	0.81(0.49-1.36)	0.45
73	hsa-miR-656-5p	Not Available				

Supplementary Table 5: Differentially expressed genes in C14MC in CaSki cell line

**Type**	**UPREGULATED**	**DOWNREGULATED**
Protein coding genes	396	593
lncRNA	51	65
processed pseudogenes	22	27
transcribed processed pseudogene	1	1
transcribed unitary pseudogene	1	4
transcribed unprocessed pseudogene	5	9
unprocessed pseudogene	6	4
miscRNA	NIL	1
mitochondrial RNA	NIL	1

Supplementary Table 6: C14MC members targeting PDK3 predicted using TargetScan

S.No	C14MC Members	Binding Sites - 3'UTR PDK3
1	hsa-miR-379	7440-7460
2	hsa-miR-381	7808-7828
3	hsa-miR-409	6491-6502,8972-8992
4	hsa-miR-494	4891-4813, 8591-8598
5	hsa-miR-539	7880-7894
6	hsa-miR-377	4791-4813
7	hsa-miR-758	6491-6502
8	hsa-miR-656	4914-4936
