# Supplementary material for: Regulation and tumor-suppressive function of the miR-379/miR-656 (C14MC) cluster in cervical cancer
Source: Mol Oncol. Author manuscript; Available in PMC 2024 Jun 10. (PMC11161731; doi:10.1002/1878-0261.13611)
Supplement: Supplementary figures and tables Legends [file EMS195193-supplement-Supplementary_figures_and_tables_Legends.pdf]

expression and subcellular localization of pyruvate dehydrogenase complex in prostate cancer. *Front Oncol*. 2022;**12**:873516.

85 Anwar S, Shamsi A, Mohammad T, Islam A, Hassan MI. Targeting pyruvate dehydrogenase kinase signaling in the development of effective cancer therapy. *Biochim Biophys Acta Rev Cancer*. 2021;**1876**(1):188568.

86 Xu J, Shi Q, Xu W, Zhou Q, Shi R, Ma Y, et al. Metabolic enzyme PDK3 forms a positive feedback loop with transcription factor HSF1 to drive chemoresistance. *Theranostics*. 2019;**9**(10):2999–3013.

Supporting information

Additional supporting information may be found online in the Supporting Information section at the end of the article.

Fig. S1. Heatmap of C14MC expression in the TCGA-CESC cohort.

Fig. S2. *In silico* analysis of *MEF2* family members in the TCGA-CESC dataset.

Fig. S3. Clinical utility of C14MC in the TCGA-CESC cohort.

Fig. S4. Gene Ontology analysis of C14MC target genes in CC.

Fig. S5. The PPIN network of C14MC-modulated genes.

Fig. S6. Gene Ontology (GO) analysis of the hub genes.

Table S1. Cell Line authentication Using GenePrint-10 System by STR Profiling.

Table S2. Antibodies and corresponding information used for performing western blotting.

Table S3. TCGA expression profile of the C14MC cluster in CC.

Table S4. C14MC member expression, ROC curve and survival analysis, performed in cervical cancer using miRNOME.

Table S5. Differentially expressed genes in the C14MCA CaSki cell line.

Table S6. C14MC members targeting *PDK3* predicted using TargetScan.

18780261, 0, Downloaded from https://febs.onlinelibrary.wiley.com/doi/10.1002/1878-0261.13611 by Kasturba Medical College, Manipal, Wiley Online Library on [06/04/2024]. See the Terms and Conditions (https://onlinelibrary.wiley.com/terms-and-conditions) on Wiley Online Library for rules of use; OA articles are governed by the applicable Creative Commons License
